# Pharmacological interventions for reducing the risk of cognitive decline and dementia after stroke/transient ischemic attack: A systematic review

**DOI:** 10.1016/j.tjpad.2026.100680

**Published:** 2026-10-12

**Authors:** Colin Rosenau, Lieke Bakker, Veerle van Gils, Sebastian Köhler, Kay Deckers

**Affiliations:** Alzheimer Centre Limburg, Department of Psychiatry and Neuropsychology, Mental Health and Neuroscience Research Institute (MHeNs), Maastricht University, Maastricht, The Netherlands

**Keywords:** Prevention, Dementia, Stroke, TIA, Pharmacological intervention

## Abstract

**Background:**

Stroke is a leading cause of death and disability worldwide. Advances in stroke care have improved survival and recovery, but it remains unclear whether pharmacological treatments can prevent long-term consequences including cognitive decline and dementia. This systematic review examined whether pharmacological interventions reduce the risk of cognitive decline, mild cognitive impairment (MCI), or dementia in adults with a history of stroke or symptomatic transient ischemic attack (TIA).

**Methods:**

We searched Medline, EMBASE, PsychINFO, and Cochrane Central from inception to January 23, 2025, for systematic reviews and meta-analyses of randomized controlled trials (RCTs). Eligible studies included adults with prior stroke or symptomatic TIA receiving pharmacological interventions, blood pressure-lowering, antithrombotic, and lipid-lowering drugs, compared with placebo, no treatment, or usual care. Primary outcomes were incident dementia, MCI, and cognitive decline assessed at least three months post-event. Methodological quality was assessed with AMSTAR 2, and certainty of evidence using GRADE. The protocol was registered in PROSPERO (CRD420251000825).

**Results:**

Of 1343 records screened, 8 systematic reviews met inclusion criteria. The most comprehensive systematic review, including all relevant RCTs, was selected for detailed data extraction. Certainty of evidence was mostly low, with a few exceptions of moderate to high certainty. Across drug classes and outcomes, pharmacological interventions generally showed no meaningful reductions in dementia, MCI, and cognitive decline over a mean follow-up of 2.0–4.9 years.

**Conclusions:**

Current evidence does not support pharmacological interventions after a cerebrovascular event for reducing risk of cognitive decline, MCI, or dementia. Further high-quality studies with longer follow-ups are needed.

## 1 Introduction

Stroke is a leading cause of death and disability worldwide, affecting approximately 100 million individuals globally, with around 12 million new cases each year [[1], [2], [3]]. Advances in acute stroke management have improved survival rates, increasing the number of individuals with stroke-related disabilities [1]. Among these, cognitive impairment and dementia are particularly common and have a significant impact on quality of life and impose a considerable burden on caregivers and healthcare systems. Stroke survivors, caregivers, and health professionals identified cognition as the top research priority relating to life after stroke [4].

Approximately 30%−50% of stroke survivors develop mild cognitive impairment (MCI) or dementia, involving long-term cognitive problems, spanning domains of memory, attention, executive function, and visuospatial skills. The risk of MCI and dementia increases with the number and severity of strokes, highlighting the importance of effective secondary prevention strategies [[5], [6], [7]]. Post-stroke cognitive decline is multifactorial and may result from underlying progression of vascular brain injury, including white matter hyperintensities (WMH) [8].

Acute stroke (period up to 12 weeks after stroke) requires acute treatment and subsequent rehabilitation to regain skills and independence. Six months after stroke, persisting deficits are more likely to reflect lasting deficits rather than temporary effects of stroke or the recovery process [9,10]. A transient ischemic attack (TIA) is a temporary reduction in blood flow to the brain that causes stroke-like symptoms that resolve within 24 h.

Several systematic reviews have previously evaluated pharmacological interventions, such as blood pressure-lowering, antithrombotic, and lipid-lowering drugs, in their ability to mitigate dementia risk through secondary stroke prevention [[11], [12], [13], [14]]. However, these reviews generally did not provide a comprehensive synthesis across multiple pharmacological classes and treatment protocols for the various cognitive outcomes.

Currently, the WHO does not provide specific guidance on pharmacological management of post-stroke cognitive decline, resulting in limited directions for clinicians and policy makers. Commissioned by the WHO, we conducted a new umbrella review to summarize the most recent evidence from systematic reviews and meta-analyses on pharmacological interventions aimed at preventing secondary stroke (blood pressure-lowering, antithrombotic, and lipid-lowering drugs) for the prevention of cognitive impairment or dementia >3 months after stroke. This review evaluates the quality and strength of available evidence, identifies gaps in current knowledge, and provides an updated evidence base which informed the second edition of the WHO guidelines on risk reduction of cognitive decline and dementia released in 2026, including stroke as a new factor.

## 2 Methods

### 2.1 Search strategy and selection criteria

This umbrella review was conducted in accordance with Chapter 8 of the *WHO Handbook for Guideline Development* (2nd edition) and PRISMA 2020 guidelines [15,16]. The protocol was registered in PROSPERO (CRD420251000825). Systematic reviews and meta-analyses published until Jan 23, 2025 were included that (1) included studies with a randomized controlled trial (RCT) design that (2) assessed the effect of pharmacological interventions (specifically blood pressure-lowering, antithrombotic, and lipid-lowering) for long-term management and prevention of secondary stroke/TIA (3) in adult individuals with an acute cerebrovascular event (stroke/TIA) (4) on a cognitive outcome (dementia, MCI, or cognitive decline) (5) measured at least 3 months post-event (i.e., in the post-acute phase). We excluded studies focusing on non-pharmacological interventions or acute stroke/TIA interventions (e.g., thrombolysis, surgical interventions), populations other than stroke/TIA survivors, pediatric populations, studies assessing outcomes unrelated to cognition or dementia, and non-systematic reviews, non-peer-reviewed studies and primary literature. No date and language restrictions were imposed. Inaccessible studies and studies in official WHO languages other than English were accessed and assessed by a WHO colleague. Full eligibility criteria can be found in Supplementary material 1.

We have searched MEDLINE (OvidSP), Embase (OVID), APA PsycINFO (EBSCO), and Cochrane Library. The search strategy was developed in consultation with a university librarian and information specialist of the WHO and comprised a combination of (masked) free text terms and controlled vocabulary covering cognitive outcomes (eg, “dementia*” or “cognition*”), cerebrovascular disease (eg, “stroke*” or “cerebrovascular”) and pharmacological interventions (eg, “intervention*” or “manag*”) for title and abstract. To ensure full coverage of the literature, specific search terms were added for blood pressure-lowering (eg,”antihypertensive”), antithrombotic (eg, “cilostazol”), and lipid-lowering drugs (eg, “statin*”). Specific search protocols are shown in Supplementary material 2.

All search hits from the different databases were imported into Covidence, followed by automatic deduplication. Based on predetermined inclusion and exclusion criteria, the study selection process was conducted in triplicate, with LB, VG, and CR each reviewing two thirds of all abstracts, with one third of overlap within each reviewer pair for cross validation. A similar approach was followed for full-text screening, now also including the reason for full-text exclusion. Disagreements between reviewers within each reviewer pair were resolved by a consensus meeting with all three reviewers. Where no consensus could be reached, a methodologist of the WHO was consulted.

### 2.2 Data extraction and management

One review included all relevant RCTs and was identified as the best-fitting review for the research question [16], from which we extracted data on the level of the primary studies, using a standardized data extraction spreadsheet with items included in consultation with the WHO. LB, VG, and CR extracted data from two thirds of all included studies, with one third of overlap between each reviewer pair for cross-validation. Disagreements were resolved with the third reviewer and, whenever necessary, with the WHO.

Extracted items included study characteristics (study name, publication year, journal, author name, and trial name), demographics (mean age, percentage female, socioeconomic position, and ethnicity whenever available), study population (baseline cognitive status, baseline stroke/TIA status, time post-event), intervention (drug, drug class, dose, duration) and control, study outcomes, and follow-up duration. Whenever the authors did not present the necessary information to be included in the review, we extracted this from related papers where possible, or we contacted the authors for additional information.

The primary outcomes were incident cognitive impairment or dementia, expressed as odds ratio (OR) with 95% confidence intervals (CI), and cognitive decline expressed as mean difference (MD) in cognitive test performance. We considered global cognition scales and validated cognitive tests (e.g., Mini-Mental State Examination (MMSE), Montreal Cognitive Assessment (MoCA), mean of combined neuropsychological domains) for determining cognitive decline as well as individual cognitive domains whenever available in ≥2 studies. Secondary outcomes included quality of life (QoL), activities of daily living (ADL), serious adverse events (SAEs), and fatal events. Findings of secondary outcomes are presented in the supplement.

### 2.3 Quality assessment of included studies

The quality of the included systematic reviews and meta-analyses was assessed using A MeaSurement Tool to Assess systematic Reviews (AMSTAR 2) to determine whether studies had any non-critical weaknesses or critical flaws. LB, VG, and CR conducted quality assessment for two thirds of all included studies, with one third overlap between each reviewer pair for cross-validation. Disagreements between reviewers within each reviewer pair were resolved with the third reviewer. All ratings are summarized in Supplementary material 3.

### 2.4 Evidence synthesis

Some of the intervention-outcome pairs discussed in the best-fitting review were also reported as such within the current review. In primary studies where the incidence of binary outcomes for multiple study arms was presented, but no effect size was reported, or whenever intervention-outcome pairs were not explicitly synthesized in the best-fitting review, we calculated ORs using random-effects Mantel-Haenszel meta-analyses from the meta package in R (version 2025.09.0 + 387). When available, we extracted data for subgroup analyses by demographics (males versus females, comparing ethnicities), drug classes or protocols, by baseline event (stroke, hemorrhagic stroke, TIA), effect lag time (post-acute: 3 months to 2 years versus long-term: >2 years), baseline cognitive status (cognitively unimpaired versus impaired), and dementia subtypes.

### 2.5 Assessment of certainty of the evidence (GRADE evidence profiles)

Using the Grading of Recommendations, Assessment, Development and Evaluations (GRADE) framework, we graded the certainty of the evidence as very low, low, moderate, or high. Certainty assessment domains included risk of bias, inconsistency, indirectness, and imprecision, which could be downgraded by up to three points depending on the degree of domain violation. In consultation with the WHO, relative effects were converted to absolute effects in GRADEpro (www.gradepro.org), with changes more than 10 cases per 1000 considered clinically important. For continuous outcomes, a MD greater than 10% of the score range (e.g., >10 points on a 0–100 scale) was considered a clinically important effect.

### 2.6 Role of the funding source

The funding source had no role in the study design, data collection, data analysis, data interpretation, in the writing of this report, or in the decision to submit the paper for publication.

## 3 Results

Overall, 1343 abstracts were screened, and 49 papers were selected for full-text screening (Fig. 1). Of these, 8 eligible systematic reviews were considered for GRADE. As the considered papers involved considerable overlap in individual RCTs, one broad systematic review covering all relevant individual RCTs was selected. This systematic review was a collaboration of the European Stroke Organisation and the European Academy of Neurology to jointly develop evidence-based clinical guidelines on the management of post-stroke cognitive impairment using GRADE [17]. The excluded reviews are listed in Supplementary material 4.

**Fig. 1:**
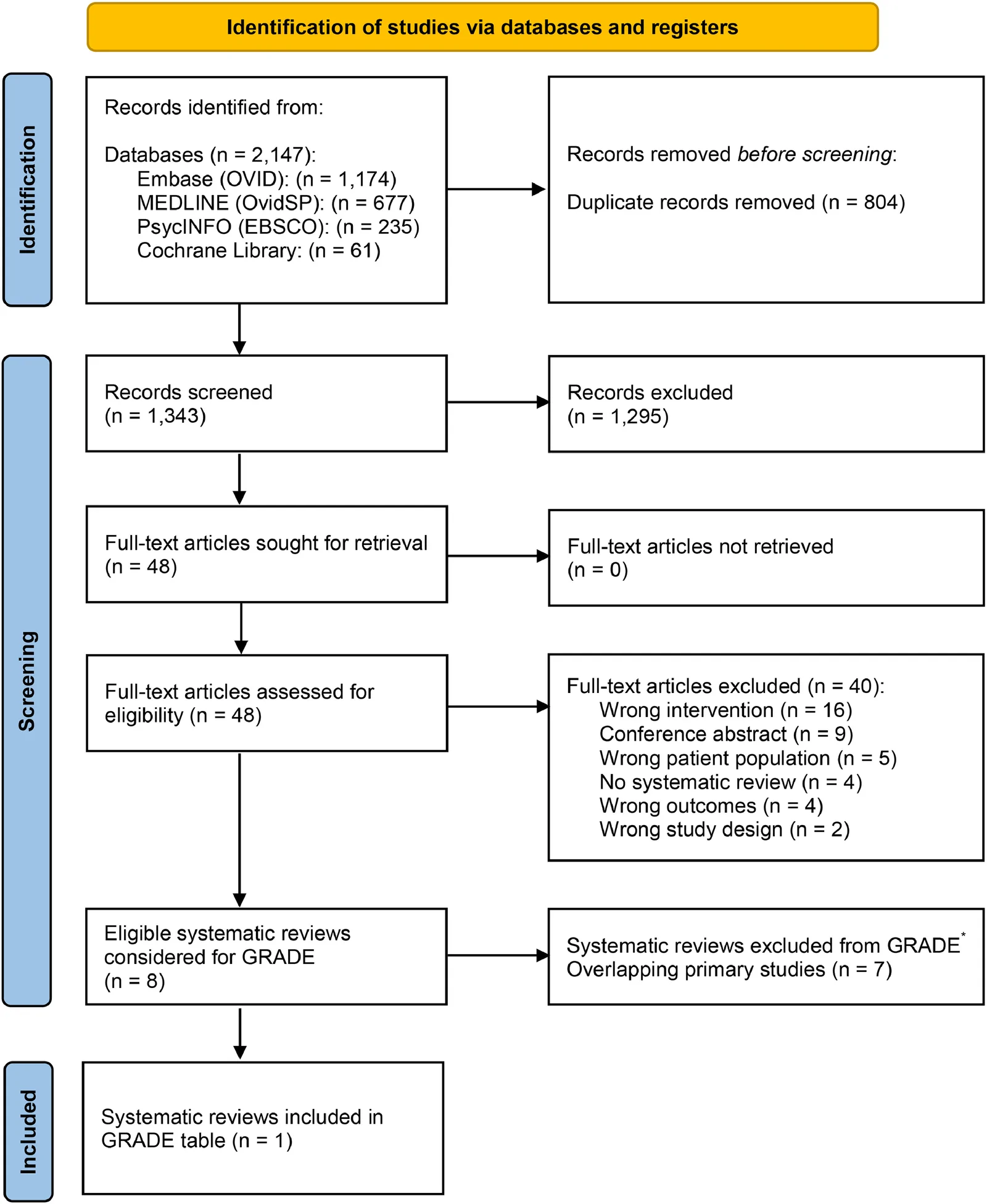
PRISMA 2020 flow diagram of review selection process.

*We excluded these reviews as all relevant primary studies matching our PICO were included in the final selected systematic review and thus only involved overlapping data.

AMSTAR 2 quality ratings of all 8 eligible systematic reviews are included in the Supplementary material 3. Except for two articles, all articles were rated as low or critically low quality. The selected systematic review by Quinn et al. (2021) was rated as critically low because it was a broad systematic review covering fifteen research questions and therefore missing detail. Despite the critically low rating, we decided to use this review as it was the only review that covered all relevant primary RCTs to our research question (n = 5) [[18], [19], [20], [21], [22]]. As not all results of the individual studies were reported in detail in the systematic review, data was extracted from the five primary RCT’s. Study characteristics are described in Table 1.

**Table 1:** Study characteristics of the included primary studies.

Author, year, country	Study name, design	Risk of Bias	Population	Sample size	Intervention	Comparator	Primary outcome(s)	Outcome measures	Follow-up measures	Result
**Intervention category: Blood pressure-lowering drugs**										
Bath et al. 2017[18] UK	PODCAST Multicenter RCT	Low risk of bias	Any stroke patients 3–7 months after stroke	Intervention: 41 Comparator: 42	Intensive SBP management (SBP < 125 mmHg) using ACE inhibitors, ARBs, β-blockers, CCBs, diuretics or others	Guideline SBP management (SBP < 140 mmHg) using guideline treatments	Cognitive decline	Mean Δ MMSE	0, 6, 18, 30, 42 months Mean follow-up: 2 years	No differences between groups in any of the cognitive outcomes.
Diener et al. 2008[19] 35 Countries or regions	PRoFESS Multicenter RCT	Low risk of bias	Ischemic stroke patients <90 days after stroke	Intervention: 10,146 Comparator: 10,186	Telmisartan (80 mg)	Placebo	Dementia Cognitive decline	Clinical impression Δ MMSE ≥ 3	1, 24 months and penultimate visit Mean follow-up: 2.5 years	No differences between groups in any of the cognitive outcomes.
Pearce et al. 2014[21] USA, Latin America, Spain	SPS3 Multicenter RCT	Some concerns (incomplete outcome data)	Lacunar stroke patients <3 months after stroke	Intervention: 1452 Comparator: 1464	Low target SBP level (SBP < 130 mmHg)	Care as usual (Higher target SBP level 130–149 mmHg)	Mild cognitive impairment	Cognitive testing (two domains ≥1.5SD or ≥2SD memory below mean)	0, 12, 24, 36, 48, 60 months Mean follow-up: 3.9 years	No differences between groups in any of the cognitive outcomes.
Tzourio et al. 2003[22] 10 countries	PROGRESS Multicenter RCT	Low risk of bias	Any stroke or TIA patients (but not SAH) <5 years after stroke	Intervention: 3051 Comparator: 3054	Perindopril (4 mg) + indapamide (2.5/2.0 mg)	Placebo	Dementia Cognitive decline	DSM IV Δ MMSE ≥ 3	Mean follow-up: 3.9 years	Blood pressure-lowering drugs resulted in decreased cognitive decline compared to placebo. No differences between groups regarding dementia.
**Intervention category: Antithrombotic drugs**										
Diener et al. 2008[19] 35 Countries or regions	PRoFESS Multicenter RCT	Low risk of bias	Ischemic stroke patients < 90 days after stroke	Intervention: 10,181 Comparator: 10,151	Low-dose acetylsalicylic acid (25 mg) + extended-release dipyridamole (200 mg)	Clopidogrel (75 mg)	Dementia Cognitive decline	Clinical impression Δ MMSE ≥ 3	1, 24 months and penultimate visit Mean follow-up: 2.5 years	No differences between groups in any of the cognitive outcomes.
Pearce et al. 2014[21] USA, Latin America, Spain	SPS3 Multicenter RCT	Some concerns (incomplete outcome data)	Lacunar stroke patients < 6 months after stroke	Intervention: 1468 Comparator: 1448	Aspirin (325 mg) + Clopidogrel (75 mg)	Aspirin (325 mg) + Placebo	Mild cognitive impairment	Cognitive testing (two domains ≥1.5SD or ≥2SD memory below mean)	0, 12, 24, 36, 48, 60 months Mean follow-up: 3.9 years	No differences between groups in any of the cognitive outcomes.
**Intervention category: Lipid-lowering drugs**										
Bath et al. 2017[18] UK	PODCAST Multicenter RCT	Low risk of bias	Ischemic stroke patients 3–7 months after stroke	Intervention: 39 Comparator: 38	Intensive LDL-C management (LDL-C < 2.0 mmol/L) using fluvastatin, simvastatin, atorvastatin, rosuvastatin, or ezetimibe	Guideline LDL-C management (LDL-C < 3.0 mmol/L) using guideline treatments	Cognitive decline	Mean Δ MMSE	0, 6, 18, 30, 42 months Mean follow-up: 2 years	No differences between groups in any of the cognitive outcomes.
Hosomi et al. 2015[20] Japan	J-STARS Multicenter RCT	Some concerns (blinding of participants)	Ischemic non-cardioembolic stroke patients < 3 years after stroke	Intervention: 793 Comparator: 785	Pravastatin (10 mg)	No statins (other lipid-lowering drugs allowed)	Dementia	DSM IIIR	2, 6, 12, 24, 36, 48, 60 months Mean follow-up: 4.9 years	No differences between groups in any of the cognitive outcomes.

Abbreviations: ACE, angiotensin-converting enzyme; ARB, angiotensin II receptor blocker; CASI, The Cognitive Abilities Screening Instrument; CCB, calcium channel blocker; DSM IV, Diagnostic and Statistical Manual of Mental Disorders; LDL-C, low-density lipoprotein cholesterol; MMSE, Mini-Mental State Examination; RCT, randomized controlled trial; SAH, subarachnoid hemorrhage; SBP, systolic blood pressure; TIA, transient ischemic attack.

For the different drug classes that were covered (blood pressure-lowering, antithrombotic, lipid-lowering), some of the studies investigated specific drug types and others aimed for stricter targets as compared to current clinical guidelines for either blood pressure or cholesterol levels by individualized drug management and were separately rated through GRADE. The primary outcomes are described in Table 2 and a visual summary of all the GRADED evidence for all included outcomes is shown in Table 3. Detailed evidence profiles (also including secondary outcomes) are included in Supplementary material 5. Subgroup analyses could not be performed due to insufficient data. Interpretations based on GRADE profiles for each drug class are described below.

**Table 2:** Summary of GRADE-rated evidence on the effect of pharmacological interventions on the risk of cognitive decline and dementia after Stroke/TIA.

Intervention/ Comparator	RCTs	Number of participants	Outcome	Effect size	Absolute effect difference	Certainty of the evidence	Summary
Blood pressure-lowering medication versus placebo	PRoFESS[19] PROGRESS[22]	Intervention: 11,675 Comparator: 11,700	Incident dementia (DSM-IV)	OR: 0.96 (CI 95% 0.86 – 1.08)	2 fewer per 1000 (from 7 fewer to 4 more)	High	Blood pressure-lowering treatment in adults that experienced a stroke or TIA makes little to no difference on dementia incidence.
Blood pressure-lowering medication versus placebo	PRoFESS[19] PROGRESS[22]	Intervention: 10,582 Comparator: 10,572	Cognitive decline (Δ MMSE ≥ 3)	OR: 0.89 (CI 95% 0.76 – 1.04)	11 fewer per 1000 (from 24 fewer to 4 more)	Low, downgraded due to inconsistency in findings between RCTs and imprecision (confidence interval including both trivial effect and important benefit)	Blood pressure-lowering treatment in adults that experienced a stroke or TIA may reduce risk of cognitive impairment compared to placebo.
Blood pressure lowering medication: stricter versus regular guidelines	SPS3[21]	Intervention: 713 Comparator: 700	MCI (≥ 1.5 SD below mean on ≥ 2 memory score)	OR: 1.03 (CI 95% 0.82 – 1.31)	6 more per 1000 (from 37 fewer to 56 more)	Low, very serious imprecision, downgraded with 2 levels due to confidence interval including both important benefit and harm	Stricter blood pressure-lowering guidelines in adults that experienced a stroke may make little to no difference on mild cognitive impairment incidence compared to regular blood pressure-lowering guidelines.
Blood pressure lowering medication: stricter versus regular guidelines	PODCAST[18]	Intervention: 41 Comparator: 42	Cognitive decline (Mean Δ MMSE)	Mean difference in MMSE: 1.2 points lower (lower means worse; CI 95% 3.3 points lower to 0.8 higher)	-	Low, very serious imprecision, downgraded with 2 levels due to very small samples size and confidence interval including both trivial benefit and important harm	Stricter blood pressure-lowering guidelines in adults that experienced a stroke may make little to no difference on cognitive decline compared to regular blood pressure-lowering guidelines.
Dual antithrombotic treatment versus care as usual	PRoFESS[19]	Intervention: 8607 Comparator: 8663	Incident dementia (DSM-IV)	OR: 1.10 (CI 95% 0.96 – 1.27)	4 more per 1000 (from 2 fewer to 11 more)	Moderate, imprecision downgraded with 1 level due to confidence interval including both trivial effect and important harm	Dual antithrombotic treatments in adults that experienced a stroke likely makes little to no difference on dementia incidence compared to monotreatment.
Dual antithrombotic treatment versus care as usual	SPS3[21]	Intervention: 721 Comparator: 692	MCI (≥ 1.5 SD below mean on ≥ 2 memory score)	OR: 0.96 (CI 95% 0.76 – 1.21)	8 fewer per 1000 (from 51 fewer to 39 more)	Low, imprecision downgraded with 2 levels due to confidence interval including both important benefit and important harm	Dual antithrombotic treatments in adults that experienced a stroke may make little to no difference on mild cognitive impairment incidence compared to monotreatment.
Dual antithrombotic treatment versus care as usual	PRoFESS[19]	Intervention: 7427 Comparator: 7622	Cognitive decline (Δ MMSE ≥ 3)	OR: 0.90 (CI 95% 0.81 – 1.00)	10 fewer per 1000 (from 19 fewer to 0 fewer)	Moderate, imprecision downgraded with 1 level due to confidence interval including both trivial effect and important harm	Dual antithrombotic treatments in adults that experienced a stroke likely makes little to no difference on cognitive decline compared to monotreatment.
Statins treatment versus no statins treatment	J-STARS[20]	Intervention: 780 Comparator: 785	Incident dementia (DSM-IIIR)	OR: 0.97 (CI 95% 0.59 – 1.60)	1 fewer per 1000 From 17 fewer to 24 more	Low, imprecision downgraded with 2 levels due to confidence interval including both important harm and important benefit	Statins treatment in adults that experienced a non-cardioembolic ischemic stroke may make little to no difference on dementia incidence compared to no statins treatment.
Lipid-lowering treatment: stricter versus regular guidelines	PODCAST[18]	Intervention: 39 Comparator: 38	Cognitive decline (Δ MMSE)	Mean difference: 1.9 points higher (higher means better; CI 95% 0.3 points lower to 4.1 points higher)	-	Low, imprecision downgraded with 2 levels due to very small sample size and due to confidence interval including both trivial effect and important benefit	Stricter lipid-lowering guidelines in adults that experienced an ischemic or hemorrhagic stroke may make little to no difference on cognitive decline compared to regular lipid-lowering guidelines.

Abbreviations: DSM, Diagnostic and Statistical Manual of Mental Disorders; MMSE, Mini-Mental State Examination; RCT, randomized controlled trial; TIA, transient ischemic attack.

**Table 3:** Visual summary of GRADED evidence.

To define minimally important difference, we used the following effect/thresholds for benefit or harm:

Binary outcomes (i.e., dementia, MCI): >10 per 1000, lower absolute effect means no meaningful effect.

Continuous outcomes (i.e., cognitive function): standardized mean difference: >10% of raw score range, lower difference means no meaningful effect.

Certainty of the evidence based on GRADE: ; ; ; .

With respect to blood pressure-lowering treatment versus placebo, a meta-analysis of the evidence from the PRoFESS and PROGRESS trials (n = 23,375, mean follow-up 2.5–3.9 years) found a pooled OR of 0.96 [95%CI 0.86–1.08] for dementia incidence (Fig. 1A), with an absolute risk difference of 2 fewer cases per 1000 persons [19,22] (Fig. 2). This indicates that treatment after stroke or TIA made little to no difference in dementia incidence. Certainty of evidence was high.

**Fig. 2:**
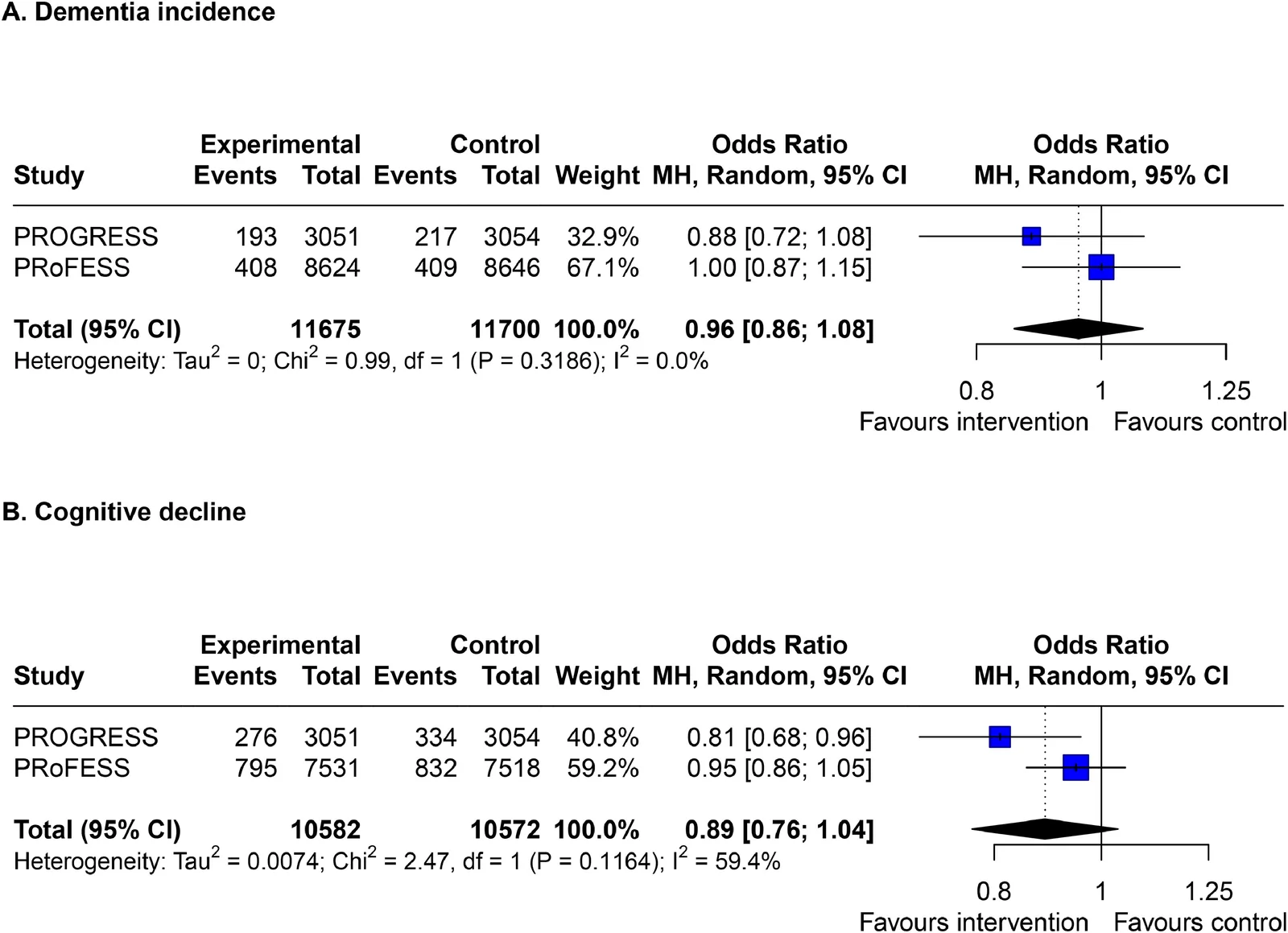
Forest plots of associations between blood pressure-lowering drugs and A. Dementia incidence and B. Cognitive decline as defined by an MMSE decline of ≥3 points.

For cognitive decline, a meta-analysis of the same RCTs (n = 21,154, mean follow-up 2.4–3.9 years) found a pooled OR of 0.89 [95%CI 0.76–1.04] (Fig. 1B, Fig. 2), with an absolute difference of 11 fewer cases per 1000 persons [19,22]. This exceeds the predefined threshold of a clinically important effect and therefore indicates that blood pressure-lowering treatment after stroke or TIA may reduce the risk of cognitive decline, but the confidence interval included a trivial effect. Certainty of evidence was low due to inconsistency and imprecision.

For stricter versus regular blood pressure targets after stroke, the SPS3 trial (n = 1413, mean follow-up 3.9 years) indicated an OR of 1.03 [95%CI 0.82–1.31] for MCI, with an absolute difference of 6 more cases per 1000 persons, suggesting that post-stroke stricter blood pressure-lowering guidelines may make little to no difference on MCI incidence compared to regular guidelines [21]. Evidence was low due to very serious imprecision. The PODCAST trial (n = 83, mean follow-up 2 years) assessed cognitive decline, and found that adhering to stricter blood pressure-lowering guidelines resulted in a mean difference of 1.2 points lower [95%CI −3.3–0.8] on the MMSE, indicating that post-stroke stricter blood pressure-lowering guidelines may make little to no difference on cognitive decline compared to regular guidelines [18]. Certainty of evidence was low due to very serious imprecision.

With respect to dual antithrombotic treatment versus monotreatment (care as usual), the PRoFESS trial (n = 17,270, mean follow-up 2.5 years) indicated an OR of 1.10 [95%CI 0.96–1.27] for dementia incidence, with an absolute difference of 4 more cases per 1000 persons [18]. This suggests that dual antithrombotic treatment after stroke likely makes little to no difference on dementia incidence, with moderate certainty due to serious imprecision. PRoFESS (n = 15,049, mean follow-up 2.5 years) additionally indicated an OR of 0.90 [95%CI 0.81–1.00] for cognitive decline, with an absolute difference of 10 fewer cases per 1000 persons. This indicated that post-stroke dual antithrombotic treatment likely makes little to no difference on cognitive decline compared to monotreatment, with moderate certainty of evidence due to imprecision.

Similarly, the SPS3 trial (n = 1413, mean follow-up 3.9 years) indicated an OR of 0.96 [95%CI 0.76–1.21] for MCI, with an absolute difference of 8 fewer cases per 1000 persons [21]. This indicates that post-stroke dual antithrombotic treatment may make little to no difference on MCI incidence compared to monotreatment. Certainty was low due to very serious imprecision.

With respect to statin treatment versus no statins, the J-STARS trial (n = 1565, mean follow-up 4.9 years) showed an OR of 0.97 [95%CI 0.59–1.60] for dementia incidence, with an absolute difference of 1 fewer cases per 1000 persons [20]. This indicated that post-stroke statin treatment may make little to no difference on dementia incidence compared to no statin treatment. Certainty of evidence was low due to very serious imprecision.

The PODCAST trial (n = 77, mean follow-up 2 years) found that adhering to stricter versus regular lipid targets after stroke resulted in a mean difference of 1.9 points higher [95%CI −0.3–4.1] on the MMSE indicating that post-stroke adherence to stricter guidelines may make little to no difference on cognitive decline compared to regular guidelines [18]. Certainty of evidence was low due to very serious imprecision.

Secondary outcomes were measured in part of the trials: PRoFESS and PODCAST reported fatal events [18,19], PRoFESS, PODCAST and J-STARS reported SAEs [[18], [19], [20]], and PODCAST reported both quality of life (EQ-VAS) and ADL (Barthel Index) [17]. GRADE profiles and interpretation are shown in Supplementary material 5. In short, PRoFESS (n = 20,332) indicated with moderate certainty that blood pressure-lowering drugs likely increase SAEs (OR=1.08, 95%CI 1.02–1.15) [18]. Similarly, J-STARS (n = 1565) indicated with moderate certainty that statins treatment likely increases SAEs (OR=1.20, 95%CI 0.95–1.53) [20]. The PODCAST trial (n = 77) showed a significant mean difference of 8.6 points higher (95%CI 1.7–15.5) on the EQ-VAS quality of life score when adhering to stricter lipid lowering guidelines as compared to regular guidelines, although this difference was not rated as clinically important and certainty was low [18]. Other pharmacological intervention and secondary outcome pairs did not show differences between the treatment groups and/or were rated as very uncertain.

## 4 Discussion

This systematic review examined whether pharmacological interventions reduce the risk of cognitive decline or dementia in adults with a history of stroke or symptomatic TIA, in addition to preventing recurrent stroke. We selected one overarching systematic review which assessed whether monitored intensive management of vascular risk factors can prevent post-stroke cognitive decline and dementia and included all five primary RCTs that were relevant to our research question and from which data were individually extracted [17]. Certainty of evidence ranged from very low to moderate. Across drug classes and outcomes, effect estimates for dementia, MCI, and cognitive decline generally showed no meaningful benefit over a mean follow-up of 2.0 to 4.9 years.

Regarding the effect of blood pressure-lowering medication versus placebo on dementia incidence in post-stroke patients, we extracted the results from the meta-analysis of Quinn et al. (2021) [17] including the PRoFESS and PROGRESS studies, concluding that there is no effect of blood pressure-lowering medication versus placebo. These results were in line with a Cochrane review by Zonneveld et al. (2018) that included the same studies [23]. Similarly, the SPS3 and PODCAST trials, in which intensive management of blood pressure was compared to care as usual, reported no differences between the groups in any of the cognitive outcomes [18,21]. It is important to note that PROGRESS did find a significant effect of blood pressure-lowering medication on dementia incidence for a subgroup without baseline cognitive impairment [22]. Additionally, a modest protective effect was found on cognitive decline, both when defined continuously by a difference in the mean MMSE score and when categorized with a cut-off MMSE score change of ≥3. Another trial, the NICE trial, assessed the effect of 6-month nimodipine treatment on cognition but was excluded in the current study due to the focus on acute stroke patients (<7 days since stroke) and short follow-up [24]. In this trial, 4.2% of the participants in the intervention group declined by three points or more on the MMSE compared to 7.2% in the control group (p = 0.15), while 8.4% versus 8.9%, respectively, improved by four points or more on the Alzheimer’s Disease Assessment Scale-Cognitive Subscale (ADAS-cog; p = 0.88). Although not significant at the predefined thresholds, small but significant cognitive improvements were observed when less stringent cut-offs were used: zero points on the MMSE (p < 0.01) and two on the ADAS-cog (p < 0.01) [23]. Nimodipine is a calcium channel blocker normally used within the first three weeks after stroke, and unlike other calcium channel blockers, is not used to treat hypertension.

In contrast to PROGRESS and NICE, PRoFESS did not show a significant effect on any of the cognitive outcomes, and even indicated an increase in SAEs when using blood pressure-lowering drugs [19]. Therefore, by combining results from PROGRESS and PRoFESS our results indicated that blood pressure-lowering medication may reduce the risk of cognitive impairment based on the defined clinically important difference, although this was not statistically significant in the meta-analysis. Differences between these trials could be due to differences in the intervention (drugs and protocols) or follow-up times (which were on average 3.9 years in PROGRESS and 2.0 years in PRoFESS). Additionally, PROGRESS, was the only study that included both stroke and TIA patients.

Regarding antithrombotics, our review included the findings of PRoFESS and SPS3 [19,21]. It must be noted that these trials are difficult to compare as the SPS3 trial used clopidogrel within their dual antithrombotic intervention [21], whereas the PRoFESS trial used the same drug in their monotreatment control group [19]. For this reason, as well as differences in outcome measures, none of the results from these studies were pooled. PRoFESS showed no effects of antiplatelet therapy on dementia incidence and cognitive decline, though trends indicate that more research is necessary to further explore the potential association with cognitive decline [19]. SPS3 also reported no difference in MCI incidence between the intervention groups and additionally reported that dual antiplatelet did not influence cognitive decline measured with the Cognitive Abilities Screening Instrument score (CASI; MD=0.14, p = 0.86) although not enough detail was reported to include the latter in the GRADE rating [21].

With respect to statins, two studies (J-STARS [20] and PODCAST [18]) were included. In the J-STARS trial, the effect of Pravastatin was compared to the use of other lipid-lowering medication (no statins), but found no effect on dementia incidence or cognition (MMSE MD=0.2, p = 0.18) [20]. A clinically important but non-significant increase was found in SAEs. In contrast, another systematic review including a meta-analysis of four observational cohort studies on post-stroke statin use was not associated with dementia, but was associated with a lower risk of cognitive impairment [14]. It is important to note that only one of these four studies included dementia as an outcome and included patients after hemorrhagic stroke for approximately four years of follow-up [25].

The PODCAST trial, which compared stricter lipid-lowering targets with guideline management, found no effect on differences in MMSE scores over a mean follow-up of two years [18]. The PODCAST trial also reported dementia incidence but too few cases occurred to be included in the GRADE profiles. Stricter lipid-lowering targets were associated with improved scores for other cognitive outcomes, including the trail making test A (attention), animal fluency (language), Stroop interference (executive functions) as well as with improved quality of life (EQ-VAS). However, it must be noted that this trial included many outcome measures and had a relatively small sample size (N = 77) [18].

Another study that was excluded from both our and Quinn et al.’s study is the SERVED trial in which guidelines for risk management targeting blood pressure, cholesterol and glucose as well as adequacy for anticoagulation were used at the same time [26]. There were no differences between the intervention and control group (usual care), and the drop-out was very high (48%).

Besides cardiovascular pharmacological interventions as targeted in the current study, interventions for the prevention of post-stroke cognitive impairment were more broadly assessed in the study of Quinn et al. (2021) covering multidomain lifestyle interventions, physical activity interventions, cognitive training interventions, and multicomponent interventions combining lifestyle and pharmacological interventions. Very few of these studies included dementia as an outcome and cognitive outcomes were very heterogeneous across studies [17]. Likewise, a substantial proportion of the studies that we excluded at the abstract screening stage investigated non-pharmacological interventions other than those of interest, including various lifestyle-based approaches (primarily physical activity and cognitive training) as well as less conventional interventions such as transcranial magnetic stimulation, acupuncture, and virtual reality-assisted rehabilitation [[27], [28], [29]]. As the primary focus of the current review is reducing the risk of post-stroke dementia and cognitive decline trough the prevention of recurrent cerebrovascular events, intervention that do not directly target this mechanism were excluded (e.g., symptomatic drugs for dementia, SSRI’s). These include drugs for acute stroke (e.g., nimodipine, citicoline), cholinesterase inhibitors (e.g., memantine), anti-depressants (e.g., selective serotonin reuptake inhibitors), and vitamin supplements (e.g., polyunsaturated fatty acids, especially omega-3 s) [[30], [31], [32], [33]]. Therefore, the lack of evidence for interventions that reduce the risk of cognitive decline or dementia by preventing recurrent cerebrovascular events does not preclude the potential effect of pharmacological intervention targeting the central nervous system through other mechanisms.

A key strength of this study is the rigor of the methodology. The extensive search strategy was developed in close consultation with the WHO, ensuring a broad coverage of the literature. Study selection and data extraction were performed independently in triplicate, enhancing the accuracy and reliability of the review process. All the included evidence originated from RCTs, strengthening causal inference and reducing the risk of bias. Furthermore, the certainty of all the evidence was systematically assessed using GRADE guidelines, providing a structured, transparent approach to evaluating the confidence in the findings and their clinical implications.

Nevertheless, several limitations inherent to the available evidence should be considered when interpreting the results. Overall, there is a very limited number of intervention studies conducted specifically in post-stroke populations, and the available evidence is characterized by substantial heterogeneity across interventions and outcome measures. Nevertheless, the included studies were described in great detail. Many studies excluded at the full-text screening phase focused on broader populations, such as vascular dementia [11], (vascular) cognitive impairment [34,35], small vessel disease [34], and hypertension [36]. In line with our findings, results were mixed and heterogeneous for these populations as well. By design, this umbrella review relied on existing systematic reviews and meta-analyses, which means that relevant primary studies not captured in prior reviews may have been missed. A considerable degree of variation across the included trials further complicated evidence synthesis, particularly with respect to the intervention protocols. For example, the intervention used in the SP3 trial (75 mg clopidogrel) overlapped with the control condition of the PRoFESS trial (clopidogrel 75 mg combined with aspirin 325 mg). Additionally, the PRoFESS trial and PROGRESS trial, which were meta-analyzed, respectively used telmisartan 80 mg and perindopril 4 mg combined with indapamide 2.5 mg as their interventions. In addition, the frequent use of care-as-usual as the control condition, which might already comprise effective standard treatment, may also have reduced contrast between study arms, further reducing sensitivity to intervention effects. Most studies reported overall dementia incidence only, without distinguishing between subtypes. Given that stroke and TIA are more strongly associated with vascular dementia, intervention effects might differ across dementia subtypes, something which we could not assess within this umbrella review. Likewise, there was insufficient evidence to conduct subgroup analyses based on stroke subtype or WMH burden. Therefore the results cannot be generalized to patients with high WMH burden or lacunar stroke, in whom vascular cognitive impairment is more prominent.

Post-stroke cognitive impairment and dementia are multifactorial processes, and the underlying mechanisms through which cardiovascular risk management may influence post-event cognition are complex [8,37]. While secondary prevention efforts may reduce the risk of cognitive decline by preventive recurrent cerebrovascular event, they may also limit the progression of vascular brain injury, including WMH, which have been associated with cognitive decline [38]. The current evidence does not allow differentiation between potential direct neuroprotective effects of pharmacological interventions and indirect effects mediated through reduced vascular brain injury. Furthermore, the relatively short follow-up duration (2.0–4.9 years) of the included studies may have been insufficient to detect long-term consequences of subclinical changes in vascular brain injury, which tend to manifest over larger time-horizons [38]. Future studies incorporating serial neuroimaging, longer follow-up, and more detailed patient phenotyping, including stroke subtype and repeated cognitive assessment, are needed to better understand the potential long-term cognitive effects of cardiovascular risk management and to identify patients most likely to benefit from these interventions.

## 5 Conclusion

Previous research has shown effectiveness in the use of post-stroke cardiovascular medications (blood pressure-lowering, antithrombotic, lipid-lowering drugs) for preventing recurrent stroke. Current evidence does not provide support for pharmacological interventions after ischemic stroke or TIA for also reducing the risk of subsequent cognitive decline or dementia. Given the limited number of studies these results should be interpreted with caution and further high-quality studies with longer follow-ups and subclinical markers of vascular brain injury are needed.

## Declaration of generative AI and AI-assisted technologies in the writing process

During the preparation of this work, the authors used ChatGPT (OpenAI) and Microsoft Copilot (Microsoft) in order to improve the language, readability, and clarity of the manuscript. After using these tools, the authors reviewed and edited the content as needed and take full responsibility for the content of the published article.

## Ethical statement

Because no individual participant data was collected or analysed in this study (only aggregated effect sizes were reported or meta-analysed), the current study was not subject to ethical approval.

## CRediT authorship contribution statement

**Colin Rosenau:** Writing – original draft, Visualization, Methodology, Formal analysis, Data curation, Conceptualization. **Lieke Bakker:** Writing – original draft, Visualization, Methodology, Formal analysis, Data curation, Conceptualization. **Veerle van Gils:** Writing – original draft, Visualization, Methodology, Formal analysis, Data curation, Conceptualization. **Sebastian Köhler:** Writing – review & editing, Supervision, Project administration, Methodology, Funding acquisition, Conceptualization. **Kay Deckers:** Writing – review & editing, Supervision, Project administration, Methodology, Funding acquisition, Conceptualization.

## Declaration of competing interest

On behalf of all authors, the corresponding author states that there is no conflict of interest.

## Data Availability

No individual participant data was collected for this study. The full study protocol can be found on PROSPERO [CRD420251000825].

## Footnotes

Supplementary material associated with this article can be found, in the online version, at doi:10.1016/j.tjpad.2026.100680.
