# Supplementary material for: Pharmacological interventions for reducing the risk of cognitive decline and dementia after stroke/transient ischemic attack: A systematic review
Source: J Prev Alzheimers Dis. 2026 Oct 12;13(10):100680. doi: 10.1016/j.tjpad.2026.100680 (PMC13682371; doi:10.1016/j.tjpad.2026.100680)
Supplement: Supplementary file 1 [file mmc1.docx]

**Supplementary materials**

**1. Full eligibility criteria**

Population (P):
Adults with a history of acute cerebrovascular event (stroke/symptomatic transient ischemic attack (TIA))

Intervention (I):
Pharmacological treatment for the long-term management and prevention of secondary cerebrovascular event. This excluded drugs which need to be acutely (often non-orally) administered within hours/days after the cerebrovascular event. The following drug classes were considered.

- Blood pressure-lowering drugs
- Antithrombotic drugs
- Lipid-lowering drugs

The categorization of the interventions considered both the drug classes described above as well as treatment protocols (i.e. stricter versus less strict blood pressure and lipid target levels).

Comparator (C):
No intervention, placebo, care as usual, or less strict target levels (in case of comparison of treatment protocols)

Outcomes (O):

Primary outcomes: cognitive outcomes ≥ 3 months post-event (to measure long-term cognitive effects):

- Incident dementia (subtypes, whenever available)
- Incident mild cognitive impairment (MCI)
- Cognitive decline based on changes in cognitive test performance. We focused on global cognition scales and validated cognitive tests (eg, Mini-Mental State Examination (MMSE), Montreal Cognitive Assessment (MoCA), mean of combined neuropsychological domains) or test assessing individual cognitive domains whenever available in ≥2 studies.

Secondary outcomes: Quality of life (QoL), activities of daily living (ADL), serious adverse events (SAEs), and fatal events

Measures of effects included relative risks (RR) for binary outcomes (dementia, MCI, SAEs, fata events) and mean differences (MDs) for continuous outcomes (cognitive tests, QoL, ADL) with 95% confidence intervals (CIs) whenever available. In some cases, a score change above a certain cut-point (as predefined in the primary studies) was operationalized as a binary outcome (eg, ≥3 points change in MMSE defined as cognitive decline)

Study Design:

Systematic reviews and meta-analyses of primary studies with a randomized controlled trial (RCT) design.

Exclusion criteria:

- Studies focusing on interventions other than pharmacological interventions
- Studies focusing on populations other than stroke/TIA survivors
- Studies including pediatric (<18 years) populations
- Studies focused solely on outcomes unrelated to cognition or dementia
- Non-systematic reviews, non-peer-reviewed studies, primary literature
- Acute stroke populations, with outcomes measured < 3 months post-stroke

Subgroups of interest:

- Analyses by demographics (males versus females, comparing ethnicities)
- Analyses by drug classes (blood pressure-lowering versus antithrombotic versus lipid-lowering drugs)
- Analyses by baseline event (ischemic stroke versus hemorrhagic stroke versus TIA)
- Analyses by effect lag time (3 months to 2 years post event versus >2 years post event)
- Analyses by baseline cognitive status (cognitively unimpaired versus impaired)
- Analyses by dementia subtypes

**2. Search strategy**

The search strategy was developed with a university librarian and information specialist of the WHO. Four databases, MEDLINE (OvidSP), Embase (OVID), APA PsycINFO (EBSCO), and Cochrane Library were searched from inception to January 22^nd^ 2025 without any language limit. The searched were conducted on January 23^rd^ 2025.

**Full search term used in MEDLINE (OvidSP) | 677 search hits**

| **Step** | **Search terms** |
| --- | --- |
| Step 1: Selection of studies assessing cognitive outcomes | ((lower* OR reduc* OR loss OR losses OR slower OR better OR improv* OR effect OR Effects OR efficac* OR affect* OR impact* OR persist* OR further OR detect* OR change OR vary OR changing OR risk OR ratio OR odds OR impact OR detriment* OR influenc* OR maintenance OR protect* OR enhanc* OR prevent* OR preserv* OR greater OR restor* OR progress* OR adverse OR beneficial OR performance* OR score* OR compensat*) ADJ6 (cognitive-measurement* OR cognitive-enhanc* OR Cognitive-improv* OR cognitive-outcome* OR cognitive-domain* OR cognitive-skill* OR cognitive-change* OR cognitive-performanc* OR neurocogniti* OR cognition* OR cognitive-function* OR cognitive-decline* OR cognitive-dysfunction* OR cognitive-impairment* OR cognitive-deficit* OR cognitive-behaviour* OR dementia* OR cognitive-abilit* OR alzheimer* OR cognitive-benefit* OR neurodegeneration OR neuro-degenerat* OR neuroprotective* OR MCI OR neurophysiologic* OR Neuropsycholog* OR executive-function* OR memory OR attention-deficit*)).ti,ab |
| Step 2: Selection of studies in individuals who had a stroke or TIA | exp stroke/ OR (Stroke* OR poststroke OR ((cerebrovascular OR cerebro-vascular OR cerebrum-vascular OR brain OR brain-vascular) adj2 (apoplexy OR accident* OR attack OR lesion OR acute-isch*em* OR insufficienc* OR arrest* OR failure* OR insult)) OR isch*emic-seizure* OR transient-isch*emi* ).ti,ab |
| Step 3: Combining Step 1 with Step 2 | 1 AND 2 |
| Step 4: Selection of studies with a pharmacological intervention (blood pressure-lowering drugs, antithrombotic drugs, and lipid-lowering drugs) | (intervention* OR treatment* OR manag* OR Pharmac* OR drug OR reperfusion OR thromb* OR “acetylsalicylic acid” OR antihyp* OR “bp lowering” OR “bp-lowering” OR “blood pressure lowering” OR statin* OR “cholesterol lowering” OR “cholesterol-lowering” OR antithrombo* OR anticoagula* OR “direct oral coagula*” OR DOAC OR antiplatelet* OR P2Y12 OR “tissue plasminogen activat*” OR tPA OR t-PA OR alteplase OR activase OR streptokinase OR tenecteplase OR reteplase OR urokinase OR prourokinase OR anistreplase OR aspirin OR “carbasalate calcium” OR apixaban OR betrixaban OR dabigatran OR rivaroxaban OR edoxaban OR warfarin OR heparin OR clopidogrel OR ticagrelor OR prasugrel OR cangrelor).ti,ab |
| Step 5: Combining Step 3 with Step 4 | 3 AND 4 |
| Step 6: Exclusion of animal studies | animals/ not (animals/ and humans/) |
| Step 7: Exclusion of irrelevant publication types | (letter or historical article or comment or editorial or news or case reports).pt. |
| Step 8: Combining Step 5 with Step 6 and 7 | 5 NOT (6 OR 7) |
| Step 9: Selection of reviews and meta-analyses | ((Meta-Analysis as Topic/ or meta analy$.tw. or metaanaly$.tw. or Meta-Analysis/ or (systematic adj (review$1 or overview$1)).tw. or exp Review Literature as Topic/ or cochrane.ab. or embase.ab. or (psychlit or psyclit).ab. or (psychinfo or psycinfo).ab. or (cinahl or cinhal).ab. or science citation index.ab. or bids.ab. or cancerlit.ab. or reference list$.ab. or bibliograph$.ab. or hand-search$.ab. or relevant journals.ab. or manual search$.ab.) or ((selection criteria.ab. or data extraction.ab.) and review/)) |
| Step 10: Combining Step 8 with Step 9 | 8 AND 9 |

**Full search term used in Embase (OVID) | 1,174 search hits**

| **Step** | **Search terms** |
| --- | --- |
| Step 1: Selection of studies assessing cognitive outcomes | ((lower* OR reduc* OR loss OR losses OR slower OR better OR improv* OR effect OR Effects OR efficac* OR affect* OR impact* OR persist* OR further OR detect* OR change OR vary OR changing OR risk OR ratio OR odds OR impact OR detriment* OR influenc* OR maintenance OR protect* OR enhanc* OR prevent* OR preserv* OR greater OR restor* OR progress* OR adverse OR beneficial OR performance* OR score* OR compensat*) ADJ6 (cognitive-measurement* OR cognitive-enhanc* OR Cognitive-improv* OR cognitive-outcome* OR cognitive-domain* OR cognitive-skill* OR cognitive-change* OR cognitive-performanc* OR neurocogniti* OR cognition* OR cognitive-function* OR cognitive-decline* OR cognitive-dysfunction* OR cognitive-impairment* OR cognitive-deficit* OR cognitive-behaviour* OR dementia* OR cognitive-abilit* OR alzheimer* OR cognitive-benefit* OR neurodegeneration OR neuro-degenerat* OR neuroprotective* OR MCI OR neurophysiologic* OR Neuropsycholog* OR executive-function* OR memory OR attention-deficit*)).ti,ab |
| Step 2: Selection of studies in individuals who had a stroke or TIA | exp stroke/ OR (Stroke* OR poststroke OR ((cerebrovascular OR cerebro-vascular OR cerebrum-vascular OR brain OR brain-vascular) adj2 (apoplexy OR accident* OR attack OR lesion OR acute-isch*em* OR insufficienc* OR arrest* OR failure* OR insult)) OR isch*emic-seizure* OR transient-isch*emi* ).ti,ab |
| Step 3: Combining Step 1 with Step 2 | 1 AND 2 |
| Step 4: Selection of studies with a pharmacological intervention (blood pressure-lowering drugs, antithrombotic drugs, and lipid-lowering drugs) | (intervention* OR treatment* OR manag* OR Pharmac* OR drug OR reperfusion OR thromb* OR “acetylsalicylic acid” OR antihyp* OR “bp lowering” OR “bp-lowering” OR “blood pressure lowering” OR statin* OR “cholesterol lowering” OR “cholesterol-lowering” OR antithrombo* OR anticoagula* OR “direct oral coagula*” OR DOAC OR antiplatelet* OR P2Y12 OR “tissue plasminogen activat*” OR tPA OR t-PA OR alteplase OR activase OR streptokinase OR tenecteplase OR reteplase OR urokinase OR prourokinase OR anistreplase OR aspirin OR “carbasalate calcium” OR apixaban OR betrixaban OR dabigatran OR rivaroxaban OR edoxaban OR warfarin OR heparin OR clopidogrel OR ticagrelor OR prasugrel OR cangrelor).ti,ab |
| Step 5: Combining Step 3 with Step 4 | 3 AND 4 |
| Step 6: Exclusion of animal studies | [animals]/lim NOT ([animals]/lim AND [humans]/lim) |
| Step 7: Exclusion of irrelevant publication types | [letter]/lim OR [conference abstract]/lim OR [conference paper]/lim OR [conference review]/lim OR [editorial]/lim OR [note]/lim OR 'preprint'/exp |
| Step 8: Combining Step 5 with Step 6 and 7 | 5 NOT (6 OR 7) |
| Step 9: Selection of reviews and meta-analyses | ((Meta-Analysis as Topic/ or meta analy$.tw. or metaanaly$.tw. or Meta-Analysis/ or (systematic adj (review$1 or overview$1)).tw. or exp Review Literature as Topic/ or cochrane.ab. or embase.ab. or (psychlit or psyclit).ab. or (psychinfo or psycinfo).ab. or (cinahl or cinhal).ab. or science citation index.ab. or bids.ab. or cancerlit.ab. or reference list$.ab. or bibliograph$.ab. or hand-search$.ab. or relevant journals.ab. or manual search$.ab.) or ((selection criteria.ab. or data extraction.ab.) and review/)) |
| Step 10: Combining Step 8 with Step 9 | 8 AND 9 |

**Full search term used in APA PsycINFO (EBSCO) | 235 search hits**

| **Step** | **Search terms** |
| --- | --- |
| Step 1: Selection of studies assessing cognitive outcomes | TI ((lower* OR reduc* OR loss OR losses OR slower OR better OR improv* OR effect OR Effects OR efficac* OR affect* OR impact* OR persist* OR further OR detect* OR change OR vary OR changing OR risk OR ratio OR odds OR impact OR detriment* OR influenc* OR maintenance OR protect* OR enhanc* OR prevent* OR preserv* OR greater OR restor* OR progress* OR adverse OR beneficial OR performance* OR score* OR compensat*) N6 (cognitive-measurement* OR cognitive-enhanc* OR Cognitive-improv* OR cognitive-outcome* OR cognitive-domain* OR cognitive-skill* OR cognitive-change* OR cognitive-performanc* OR neurocogniti* OR cognition* OR cognitive-function* OR cognitive-decline* OR cognitive-dysfunction* OR cognitive-impairment* OR cognitive-deficit* OR cognitive-behaviour* OR dementia* OR cognitive-abilit* OR alzheimer* OR cognitive-benefit* OR neurodegeneration OR neuro-degenerat* OR neuroprotective* OR MCI OR neurophysiologic* OR Neuropsycholog* OR executive-function* OR memory OR attention-deficit*)) OR AB ((lower* OR reduc* OR loss OR losses OR slower OR better OR improv* OR effect OR Effects OR efficac* OR affect* OR impact* OR persist* OR further OR detect* OR change OR vary OR changing OR risk OR ratio OR odds OR impact OR detriment* OR influenc* OR maintenance OR protect* OR enhanc* OR prevent* OR preserv* OR greater OR restor* OR progress* OR adverse OR beneficial OR performance* OR score* OR compensat*) N6 (cognitive-measurement* OR cognitive-enhanc* OR Cognitive-improv* OR cognitive-outcome* OR cognitive-domain* OR cognitive-skill* OR cognitive-change* OR cognitive-performanc* OR neurocogniti* OR cognition* OR cognitive-function* OR cognitive-decline* OR cognitive-dysfunction* OR cognitive-impairment* OR cognitive-deficit* OR cognitive-behaviour* OR dementia* OR cognitive-abilit* OR alzheimer* OR cognitive-benefit* OR neurodegeneration OR neuro-degenerat* OR neuroprotective* OR MCI OR neurophysiologic* OR Neuropsycholog* OR executive-function* OR memory OR attention-deficit*)) |
| Step 2: Selection of studies in individuals who had a stroke or TIA | DE "Cerebrovascular Accidents" OR TI (Stroke* OR poststroke OR ((cerebrovascular OR cerebro-vascular OR cerebrum-vascular OR brain OR brain-vascular) N2 (apoplexy OR accident* OR attack OR lesion OR acute-isch*em* OR insufficienc* OR arrest* OR failure* OR insult)) OR isch*emic-seizure* OR transient-isch*emi* ) OR AB (Stroke* OR poststroke OR ((cerebrovascular OR cerebro-vascular OR cerebrum-vascular OR brain OR brain-vascular) N2 (apoplexy OR accident* OR attack OR lesion OR acute-isch*em* OR insufficienc* OR arrest* OR failure* OR insult)) OR isch*emic-seizure* OR transient-isch*emi*) |
| Step 3: Combining Step 1 with Step 2 | S1 AND S2 |
| Step 4: Selection of studies with a pharmacological intervention (blood pressure-lowering drugs, antithrombotic drugs, and lipid-lowering drugs) | TI(intervention* OR treatment* OR manag* OR pharmac* OR drug OR antiplatelet OR anticoagulants OR antihypertensive OR antithrombotic OR statin* OR aspirin OR clopidogrel OR plavix OR ticlopidine OR cilostazol OR Tissue Plasminogen Activator OR Alteplase OR Activase OR Streptokinase OR Tenecteplase) OR AB (intervention* OR treatment* OR manag* OR pharmac* OR drug OR antiplatelet OR anticoagulants OR antihypertensive OR antithrombotic OR statin* OR aspirin OR clopidogrel OR plavix OR ticlopidine OR cilostazol OR Tissue Plasminogen Activator OR Alteplase OR Activase OR Streptokinase OR Tenecteplase) OR AB(intervention* OR treatment* OR manag* OR pharmac* OR drug OR antiplatelet OR anticoagulants OR antihypertensive OR antithrombotic OR statin* OR aspirin OR clopidogrel OR plavix OR ticlopidine OR cilostazol OR Tissue Plasminogen Activator OR Alteplase OR Activase OR Streptokinase OR Tenecteplase) OR AB (intervention* OR treatment* OR manag* OR pharmac* OR drug OR antiplatelet OR anticoagulants OR antihypertensive OR antithrombotic OR statin* OR aspirin OR clopidogrel OR plavix OR ticlopidine OR cilostazol OR Tissue Plasminogen Activator OR Alteplase OR Activase OR Streptokinase OR Tenecteplase) |
| Step 5: Combining Step 3 with Step 4 | S3 AND S4 |
| Step 6: Exclusion of animal studies | (DE "Animals" OR DE "Animal Limb" OR DE "Animal Offspring" OR DE "Female Animals" OR DE "Infants (Animal)" OR DE "Invertebrates" OR DE "Male Animals" OR DE "Pets" OR DE "Service Animals" OR DE "Species Differences" OR DE "Vertebrates") |
| Step 7: Combining Step 5 with Step 6 | S5 NOT (S6 ) |
| Step 8: Selection of reviews and meta-analyses | (DE "Meta Analysis") OR (DE "Systematic Review") OR (DE "Literature Review") OR TX (meta N3 analy*) OR TX (synthes* N3 (literature* OR research* OR studies OR data)) OR TX ((data N2 pool*) AND studies) OR TX metaanaly* OR TI (review* OR overview*) OR TX (pooled AND analys*) OR AB (medline OR medlars OR Embase OR cinahl OR scisearch OR psychlit OR psyclit OR cinhal OR cancerlit OR cochrane OR bids OR pubmed OR ovid) OR AB ((hand OR manual OR database* OR computer*) N2 search*) OR AB (electronic N2 (database* OR "data base" OR "data bases")) |
| Step 9: Combining Step 7 with Step 8 | S7 AND S8 |

**Full search term used in Cochrane Library | 61 search hits**

| **Step** | **Search terms** |
| --- | --- |
| Step 1: Selection of studies assessing cognitive outcomes | ((lower* OR reduc* OR loss OR losses OR slower OR better OR improv* OR effect OR Effects OR efficac* OR affect* OR impact* OR persist* OR further OR detect* OR change OR vary OR changing OR risk OR ratio OR odds OR impact OR detriment* OR influenc* OR maintenance OR protect* OR enhanc* OR prevent* OR preserv* OR greater OR restor* OR progress* OR adverse OR beneficial OR performance* OR score* OR compensat*) NEAR/6 (cognitive-measurement* OR cognitive-enhanc* OR Cognitive-improv* OR cognitive-outcome* OR cognitive-domain* OR cognitive-skill* OR cognitive-change* OR cognitive-performanc* OR neurocogniti* OR cognition* OR cognitive-function* OR cognitive-decline* OR cognitive-dysfunction* OR cognitive-impairment* OR cognitive-deficit* OR cognitive-behaviour* OR dementia* OR cognitive-abilit* OR alzheimer* OR cognitive-benefit* OR neurodegeneration OR neuro-degenerat* OR neuroprotective* OR MCI OR neurophysiologic* OR Neuropsycholog* OR executive-function* OR memory OR attention-deficit*)) |
| Step 2: Selection of studies with a pharmacological intervention (blood pressure-lowering drugs, antithrombotic drugs, and lipid-lowering drugs) | (intervention* OR treatment* OR manag* OR Pharmac* OR drug OR reperfusion OR thromb* OR “acetylsalicylic acid” OR antihyp* OR “bp lowering” OR “bp-lowering” OR “blood pressure lowering” OR statin* OR “cholesterol lowering” OR “cholesterol-lowering” OR antithrombo* OR anticoagula* OR “direct oral coagula*” OR DOAC OR antiplatelet* OR P2Y12 OR “tissue plasminogen activat*” OR tPA OR t-PA OR alteplase OR activase OR streptokinase OR tenecteplase OR reteplase OR urokinase OR prourokinase OR anistreplase OR aspirin OR “carbasalate calcium” OR apixaban OR betrixaban OR dabigatran OR rivaroxaban OR edoxaban OR warfarin OR heparin OR clopidogrel OR ticagrelor OR prasugrel OR cangrelor):ti,ab |
| Step 3: Combining Step 1 with Step 2 | #1 AND #2 |
| Step 4: Selection of studies in individuals who had a stroke or TIA | [mh "stroke"] OR (Stroke* OR poststroke OR ((cerebrovascular OR cerebro-vascular OR cerebrum-vascular OR brain OR brain-vascular) NEAR/2 (apoplexy OR accident* OR attack OR lesion OR acute-isch*em* OR insufficienc* OR arrest* OR failure* OR insult)) OR isch*emic-seizure* OR transient-isch*emi* ):ti,ab |
| Step 5: Combining Step 3 with Step 4 | #3 AND #4 |
| Step 6: Selection of Cochrane Reviews | #5 (in Cochrane Reviews) |

**3. Quality assessment of included systematic reviews and meta-analyses using A MeaSurement Tool to Assess Systematic Reviews (AMSTAR 2)**

| **Author, year** | **Meta-analysis** | **1** | **2** | **3** | **4** | **5** | **6** | **7** | **8** | **9** | **10** | **11** | **12** | **13** | **14** | **15** | **16** | **Quality rating** |
| --- | --- | --- | --- | --- | --- | --- | --- | --- | --- | --- | --- | --- | --- | --- | --- | --- | --- | --- |
| Alexander, 2022 | No | No | No | No | Partial yes | Yes | No | No | Partial yes | No | No | NA | NA | No | No | NA | Yes | **Critically Low** |
| Jongstra, 2016 | Yes | Yes | Yes | No | Yes | Yes | Yes | Yes | Yes | Yes | Yes | NA | NA | Yes | Yes | NA | Yes | **High** |
| Kwan,  2022 | No | Yes | Yes | No | Yes | Yes | Yes | Yes | Yes | Yes | Yes | NA | NA | Yes | Yes | NA | Yes | **High** |
| Levine,  2011 | No | No | No | No | Partial yes | No | No | No | Partial yes | No | No | NA | NA | No | No | NA | Yes | **Critically Low** |
| Quinn, 2021* | Yes | Yes | No | No | Partial yes | Yes | Yes | No | No | Yes | No | NA | NA | Yes | No | NA | Yes | **Critically Low** |
| Rouch,  2025 | No | No | No | No | Partial yes | No | No | No | Yes | Yes | No | NA | NA | No | No | NA | Yes | **Critically Low** |
| Yang,  2020 | Yes | Yes | Yes | No | Partial yes | Yes | Yes | No | Yes | Partial yes | No | Yes | Yes | Yes | Yes | Yes | Yes | **Low** |
| Zonneveld, 2018 | Yes | Yes | Yes | No | Yes | Yes | Yes | Yes | Yes | Yes | No | No | Yes | Yes | Yes | NA | Yes | **Low** |

*Selected study for the systematic review.

**4. List of Studies Excluded at Full-Text Screening Stage (N = 7), with Corresponding Reasons**

| **Study** | **Reason for exclusion** | **Comment** |
| --- | --- | --- |
| Alexander P, Visagan S, Jawhar S, Kare A, Issa N, Issa R, Jawhar A, Thomas S, Gorantla V. Antiplatelets and Vascular Dementia: A Systematic Review. J Aging Res. 2022 Sep 19;2022:9780067. doi: 10.1155/2022/9780067. PMID: 36245899; PMCID: PMC9553722.  **AMSTAR II – Critically Low Quality** | All relevant primary studies also included in other reviews | All relevant primary studies also covered in Quinn et al. |
| Jongstra S, Harrison JK, Quinn TJ, Richard E. Antihypertensive withdrawal for the prevention of cognitive decline. Cochrane Database Syst Rev. 2016 Nov 1;11(11):CD011971. doi: 10.1002/14651858.CD011971.pub2. PMID: 27802359; PMCID: PMC6465000.  **AMSTAR II – High** | All relevant primary studies also included in other reviews | All relevant primary studies also covered in Quinn et al. |
| Kwan J, Hafdi M, Chiang LLW, Myint PK, Wong LS, Quinn TJ. Antithrombotic therapy to prevent cognitive decline in people with small vessel disease on neuroimaging but without dementia. Cochrane Database Syst Rev. 2022 Jul 14;7(7):CD012269. doi: 10.1002/14651858.CD012269.pub2. PMID: 35833913; PMCID: PMC9281623.  **AMSTAR II – High** | All relevant primary studies also included in other reviews | All relevant primary studies also covered in Quinn et al. |
| Levine DA, Langa KM. Vascular cognitive impairment: disease mechanisms and therapeutic implications. Neurotherapeutics. 2011 Jul;8(3):361-73. doi: 10.1007/s13311-011-0047-z. PMID: 21556678; PMCID: PMC3167237.  **AMSTAR II – Critically Low Quality** | All relevant primary studies also included in other reviews | All relevant primary studies also covered in Quinn et al. |
| Rouch L, Cestac P, Hanon O, Cool C, Helmer C, Bouhanick B, Chamontin B, Dartigues JF, Vellas B, Andrieu S. Antihypertensive drugs, prevention of cognitive decline and dementia: a systematic review of observational studies, randomized controlled trials and meta-analyses, with discussion of potential mechanisms. CNS Drugs. 2015 Feb;29(2):113-30. doi: 10.1007/s40263-015-0230-6. PMID: 25700645.  **AMSTAR II – Critically Low Quality** | All relevant primary studies also included in other reviews | All relevant primary studies also covered in Quinn et al. |
| Yang Z, Wang H, Edwards D, Ding C, Yan L, Brayne C, Mant J. Association of blood lipids, atherosclerosis and statin use with dementia and cognitive impairment after stroke: A systematic review and meta-analysis. Ageing Res Rev. 2020 Jan;57:100962. doi: 10.1016/j.arr.2019.100962. Epub 2019 Sep 7. PMID: 31505259.  **AMSTAR II – Low Quality** | All relevant primary studies also included in other reviews | All relevant primary studies also covered in Quinn et al. |
| Zonneveld TP, Richard E, Vergouwen MD, Nederkoorn PJ, de Haan R, Roos YB, Kruyt ND. Blood pressure-lowering treatment for preventing recurrent stroke, major vascular events, and dementia in patients with a history of stroke or transient ischaemic attack. Cochrane Database Syst Rev. 2018 Jul 19;7(7):CD007858. doi: 10.1002/14651858.CD007858.pub2. PMID: 30024023; PMCID: PMC6513249.  **AMSTAR II – Low Quality** | All relevant primary studies also included in other reviews | All relevant primary studies also covered in Quinn et al. |

**5. Detailed GRADE tables**

**GRADE table 1: Blood pressure-lowering treatment vs placebo in adults that experienced a stroke or TIA**.

Population: Adults that experienced a stroke or TIA

Intervention: Blood pressure-lowering treatment

Comparator: Placebo

References:

Diener HC, Sacco RL, Yusuf S, Cotton D, Ounpuu S, Lawton WA, Palesch Y, Martin RH, Albers GW, Bath P, Bornstein N, Chan BP, Chen ST, Cunha L, Dahlöf B, De Keyser J, Donnan GA, Estol C, Gorelick P, Gu V, Hermansson K, Hilbrich L, Kaste M, Lu C, Machnig T, Pais P, Roberts R, Skvortsova V, Teal P, Toni D, VanderMaelen C, Voigt T, Weber M, Yoon BW; Prevention Regimen for Effectively Avoiding Second Strokes (PRoFESS) study group. Effects of aspirin plus extended-release dipyridamole versus clopidogrel and telmisartan on disability and cognitive function after recurrent stroke in patients with ischaemic stroke in the Prevention Regimen for Effectively Avoiding Second Strokes (PRoFESS) trial: a double-blind, active and placebo-controlled study. Lancet Neurol. 2008 Oct;7(10):875-84. doi: 10.1016/S1474-4422(08)70198-4. Epub 2008 Aug 29. Erratum in: Lancet Neurol. 2008 Nov;7(11):985. PMID: 18757238; PMCID: PMC2772657.

Tzourio C, Anderson C, Chapman N, Woodward M, Neal B, MacMahon S, Chalmers J; PROGRESS Collaborative Group. Effects of blood pressure lowering with perindopril and indapamide therapy on dementia and cognitive decline in patients with cerebrovascular disease. Arch Intern Med. 2003 May 12;163(9):1069-75. doi: 10.1001/archinte.163.9.1069. PMID: 12742805.

| **Certainty assessment** | | | | | | | **№ of patients** | | **Effect** | | **Certainty^1^** | **Plain language summary^2^** |
| --- | --- | --- | --- | --- | --- | --- | --- | --- | --- | --- | --- | --- |
| **№ of studies** | **Study design** | **Risk of bias** | **Inconsistency** | **Indirectness** | **Imprecision** | **Other considerations** | **Interventions** | **Control** | **Relative  (95% CI)** | **Absolute  (95% CI)** |  |  |
| Outcome 1 (binary): Incident dementia (based on DSM-IV) - Minimally important difference: 10 per 1000 | | | | | | | | | | | | |
| 2 | RCT (PROGRESS, PRoFESS) | Not serious | Not serious | Not serious | Not serious | - | 601/11,675 (5.1%) | 626/11,700 (5.4%) | *Odds ratio: 0.96 (CI 95% 0.86 – 1.08)  Based on data from 23,375 participants in 2 RCTs | **2 fewer** per 1,000  (from 7 fewer to 4 more) | ⨁⨁⨁⨁ (High) | Blood pressure-lowering treatment in adults that experienced a stroke or TIA makes little to no difference on dementia incidence compared to placebo. |
| Outcome 2 (binary): Cognitive decline (based on Δ MMSE ≥ 3) - Minimally important difference: 10 per 1000 | | | | | | | | | | | | |
| 2 | RCT (PROGRESS, PRoFESS) | Not serious | Serious  Downgraded with 1 level due to differences in findings (significant in one RCT, non-significant in the other) | Not serious | Serious  Downgraded 1 level due to confidence interval including both trivial effect and important benefit | - | 1,071/10,582 (10.1%) | 1,166/10,572 (11.0%) | *Odds ratio: 0.89 (CI 95% 0.76 – 1.04)  Based on data from 21,154 participants in 2 RCTs | **11 fewer** per 1,000  (from 24 fewer to 4 more) | ⨁⨁◯◯ (Low) | Blood pressure-lowering treatment in adults that experienced a stroke or TIA may reduce risk of cognitive impairment compared to placebo. |
| Outcome 3 (binary): Fatal events** - Minimally important difference: 10 per 1000 | | | | | | | | | | | | |
| 1 | RCT (PRoFESS) | Not serious | Not serious | Not serious | Serious  Downgraded with 1 level due to confidence interval including both trivial effect and important benefit | - | 1,367 per 10,146 (13.5%) | 1,463 per 10,186 (14.4%) | Hazard ratio: 0.94 (0.87, 1.01) | **8 fewer** per 1,000  (from 17 fewer to 1 more) | ⨁⨁⨁◯ (Moderate) | Blood pressure-lowering treatment in adults that experienced a stroke or TIA likely makes little to no difference on fatal events compared to placebo. |
| Outcome 4 (binary): All SAEs*** - Minimally important difference: 10 per 1000 | | | | | | | | | | | | |
| 1 | RCT  (PRoFESS) | Not serious | Not serious | Not serious | Serious  Downgraded with 1 level due to confidence interval including both trivial effect and important harm | - | 2,812 of 10,146 (27.7%) | 2,661 of 10,186 (26.1%) | *Odds ratio: 1.08 (CI 95% 1.02 – 1.15)  Based on data from 20,332 participants in 1 RCT | **15 more** per 1,000  (from 4 more to 28 more) | ⨁⨁⨁◯ (Moderate) | Blood pressure-lowering treatment in adults that experienced a stroke or TIA likely increases SAEs compared to placebo. |

*Odds ratio determined by self-conducted random-effects Mantel-Haenszel meta-analysis.

**Cause of death: cardiovascular causes, recurrent stroke, myocardial infarction, new or worsening heart failure.

***SAEs: cardiac disorders, gastrointestinal disorders, general disorders, hepatobiliary disorders, infections, injury/poisoning, metabolism/nutrition disorders, musculoskeletal disorders, neoplasms, nervous system disorders, psychiatric disorders, renal and urinary disorders, respiratory and thoracic disorders, vascular disorders.

^1^4 categories of certainty of evidence: ⨁⨁⨁⨁ (High), ⨁⨁⨁◯ (Moderate), ⨁⨁◯◯ (Low), ⨁◯◯◯ (Very low). Examples are provided in the table.

^2^For references regarding plan language summaries, please see: <https://training.cochrane.org/sites/training.cochrane.org/files/public/uploads/Nancy%20Santesso%20slides.pdf>; <https://www.sciencedirect.com/science/article/pii/S0895435619304160>

Abbreviations: CI, confidence interval; DSM IV, Diagnostic and Statistical Manual of Mental Disorders Revision IV; MMSE, Mini-Mental State Examination; RCT, randomized controlled trial; SAE, serious adverse event ; TIA, transient ischaemic attack

**GRADE table 2: Stricter blood pressure-lowering guidelines vs regular blood pressure-lowering guidelines in adults that experienced a stroke.**

Population: Adults that experienced a stroke

Intervention: Strict blood pressure-lowering guidelines

Comparator: Regular blood pressure-lowering guidelines

References:

Bath PM, Scutt P, Blackburn DJ, Ankolekar S, Krishnan K, Ballard C, Burns A, Mant J, Passmore P, Pocock S, Reckless J, Sprigg N, Stewart R, Wardlaw JM, Ford GA; PODCAST Trial Investigators. Intensive versus Guideline Blood Pressure and Lipid Lowering in Patients with Previous Stroke: Main Results from the Pilot 'Prevention of Decline in Cognition after Stroke Trial' (PODCAST) Randomised Controlled Trial. PLoS One. 2017 Jan 17;12(1):e0164608. doi: 10.1371/journal.pone.0164608. PMID: 28095412; PMCID: PMC5240987.

Pearce LA, McClure LA, Anderson DC, Jacova C, Sharma M, Hart RG, Benavente OR; SPS3 Investigators. Effects of long-term blood pressure lowering and dual antiplatelet treatment on cognitive function in patients with recent lacunar stroke: a secondary analysis from the SPS3 randomised trial. Lancet Neurol. 2014 Dec;13(12):1177-85. doi: 10.1016/S1474-4422(14)70224-8. Epub 2014 Oct 23. PMID: 25453457; PMCID: PMC4284947.

| **Certainty assessment** | | | | | | | **№ of patients** | | **Effect** | | | **Certainty^1^** | **Plain language summary^2^** |
| --- | --- | --- | --- | --- | --- | --- | --- | --- | --- | --- | --- | --- | --- |
| **№ of studies** | **Study design** | **Risk of bias** | **Inconsistency** | **Indirectness** | **Imprecision** | **Other considerations** | **Interventions** | **Control** | **Relative  (95% CI)** | **Absolute  (95% CI)** | |  |  |
| Outcome 1 (binary): Mild cognitive impairment (≥ 1.5 SD below mean on ≥ 2 memory score) - Minimally important difference: 10 per 1000 | | | | | | | | | | | | | |
| 1 | RCT (SPS3) | Not serious | Not serious | Not serious | Very serious  Downgraded with 2 levels due to confidence interval including both important benefit and important harm | - | 192/713 (26.9%) | 184/700 (26.3%) | *Odds ratio: 1.03 (CI 95% 0.82 – 1.31  Based on data from 1,413 participants in 1 RCT | | **6 more** per 1,000  (from 37 fewer to 56 more) | ⨁⨁◯◯ (Low) | Stricter blood pressure-lowering guidelines in adults that experienced a stroke may make little to no difference on mild cognitive impairment incidence compared to regular blood pressure-lowering guidelines. |
| Outcome 2 (continuous): Cognitive decline (Mean Δ MMSE, n = 1) - Minimally important difference: 3 points MD | | | | | | | | | | | | | |
| 1 | RCT (PODCAST) | Not serious | Not serious | Not serious | Very serious  Downgraded with 2 levels due to very small sample size and confidence interval including both trivial effect and important benefit |  | Group size = 41 | Group size = 42 | Mean difference in MMSE: **1.2 points lower** (lower means worse; CI 95% 3.3 points lower to 0.8 higher) | | | ⨁⨁◯◯ (Low) | Stricter blood pressure-lowering guidelines in adults that experienced a stroke may make little to no difference on cognitive decline compared to regular blood pressure-lowering guidelines. |
| Outcome 3 (continuous): Quality of life (based on Δ EQ-VAS) - Minimally important difference: 10 points MD | | | | | | | | | | | | | |
| 1 | RCT (PODCAST) | Not serious | Not serious | Not serious | Very serious  Downgraded with 2 levels due to very small sample size and due to confidence interval including trivial effect and important benefit |  | Group size = 41 | Group size = 42 | Mean difference: **4 points lower** (lower means worse; CI 95% 10.7 points lower to 2.7 higher)  Based on data from 83 participants in 1 RCT | | | ⨁⨁◯◯ (Low) | Stricter blood pressure-lowering guidelines in adults that experienced a stroke may make little to no difference on quality of life compared to regular blood pressure-lowering guidelines. |
| Outcome 4 (continuous): ADL (based on Δ Barthel Index) - Minimally important difference: 10 points MD | | | | | | | | | | | | | |
| 1 | RCT  (PODCAST) | Not serious | Not serious | Not serious | Yes  Downgraded with 2 levels due to very small sample size | - | Group size = 41 | Group size = 42 | Mean difference: **2.1 points lower** (lower means worse; CI 95% 9.6 points lower to 5.4 higher)  Based on data from 83 participants in 1 RCT | | | ⨁⨁◯◯ (Low) | Stricter blood pressure-lowering guidelines in adults that experienced a stroke may make little to no difference on ADL compared to regular blood pressure-lowering guidelines. |
| Outcome 5 (binary): Fatal events** - Minimally important difference: 10 per 1000 | | | | | | | | | | | | | |
| 1 | RCT (PODCAST) | Not serious | Not serious | Not serious | Yes  Downgraded with 3 levels due to very small sample size and due to confidence interval including important benefit and important harm | - | 4 /41   (9.8%) | 3/42 (7.1%) | *Odds ratio: 1.41 (CI 95% 0.29 – 6.71)  Based on data from 83 participants in 1 RCT | | **26 more** per 1,000  (from 50 fewer to 69 more) | ⨁◯◯◯ (Very low) | The evidence is very uncertain about the effect of stricter blood pressure-lowering guidelines in adults that experienced a stroke on fatal events compared to regular blood pressure-lowering guidelines. |
| Outcome 6 (binary): All SAEs*** – Minimally important difference: 10 per 1000 | | | | | | | | | | | | | |
| 1 | RCT  (PODCAST) | Not serious | Not serious | Not serious | Very serious  Downgraded with 3 levels due to very small sample size and due to confidence interval including important benefit and important harm | - | 18/41 (43.9%) | 22/42 (52.4%) | *Odds ratio: 0.71 (CI 95% 0.30 – 1.69)  Based on data from 83 participants in 1 RCT | | **85 fewer** per 1,000  (from 276 fewer to 126 more) | ⨁◯◯◯ (Very low) | The evidence is very uncertain about the effect of stricter blood pressure-lowering guidelines in adults that experienced a stroke on SAEs compared to regular blood pressure-lowering guidelines. |

*Odds ratio determined by self-conducted random-effects Mantel-Haenszel meta-analysis.

**Cause of death: dementia, stroke, sudden cardiac death, death unattended, tumour – malignant, pneumonia.

***SAEs: cardiovascular, genotourinary, gastrointestinal, haematological, metabolic, musculoskeletal, nervous system, respiratory, other

^1^4 categories of certainty of evidence: ⨁⨁⨁⨁ (High), ⨁⨁⨁◯ (Moderate), ⨁⨁◯◯ (Low), ⨁◯◯◯ (Very low). Examples are provided in the table.

^2^ For references regarding plan language summaries, please see: <https://training.cochrane.org/sites/training.cochrane.org/files/public/uploads/Nancy%20Santesso%20slides.pdf>; <https://www.sciencedirect.com/science/article/pii/S0895435619304160>

Abbreviations: ADL, activities daily living; CASI, The Cognitive Abilities Screening Instrument; CI, confidence interval; DSM IV, Diagnostic and Statistical Manual of Mental Disorders Revision IV; EQ-VAS, EuroQol vidual analogue; MMSE, Mini-Mental State Examination; RCT, randomized controlled trial; SAE, serious adverse event ; SD, standard deviation

**GRADE table 3:. Dual Antithrombotic treatment vs care as usual (monotreatment) in adults that experienced a stroke**

Population: Adults that experienced a stroke

Intervention: Antithrombotic treatment

Comparator: care as usual (monotreatment)

References:

Diener HC, Sacco RL, Yusuf S, Cotton D, Ounpuu S, Lawton WA, Palesch Y, Martin RH, Albers GW, Bath P, Bornstein N, Chan BP, Chen ST, Cunha L, Dahlöf B, De Keyser J, Donnan GA, Estol C, Gorelick P, Gu V, Hermansson K, Hilbrich L, Kaste M, Lu C, Machnig T, Pais P, Roberts R, Skvortsova V, Teal P, Toni D, VanderMaelen C, Voigt T, Weber M, Yoon BW; Prevention Regimen for Effectively Avoiding Second Strokes (PRoFESS) study group. Effects of aspirin plus extended-release dipyridamole versus clopidogrel and telmisartan on disability and cognitive function after recurrent stroke in patients with ischaemic stroke in the Prevention Regimen for Effectively Avoiding Second Strokes (PRoFESS) trial: a double-blind, active and placebo-controlled study. Lancet Neurol. 2008 Oct;7(10):875-84. doi: 10.1016/S1474-4422(08)70198-4. Epub 2008 Aug 29. Erratum in: Lancet Neurol. 2008 Nov;7(11):985. PMID: 18757238; PMCID: PMC2772657.

Pearce LA, McClure LA, Anderson DC, Jacova C, Sharma M, Hart RG, Benavente OR; SPS3 Investigators. Effects of long-term blood pressure lowering and dual antiplatelet treatment on cognitive function in patients with recent lacunar stroke: a secondary analysis from the SPS3 randomised trial. Lancet Neurol. 2014 Dec;13(12):1177-85. doi: 10.1016/S1474-4422(14)70224-8. Epub 2014 Oct 23. PMID: 25453457; PMCID: PMC4284947.

| **Certainty assessment** | | | | | | | **№ of patients** | | **Effect** | | **Certainty^1^** | **Plain language summary^2^** |
| --- | --- | --- | --- | --- | --- | --- | --- | --- | --- | --- | --- | --- |
| **№ of studies** | **Study design** | **Risk of bias** | **Inconsistency** | **Indirectness** | **Imprecision** | **Other considerations** | **Interventions** | **Control** | **Relative  (95% CI)** | **Absolute  (95% CI)** |  |  |
| Outcomes 1 (binary): Incident dementia (based on DSM-IV) - Minimally important difference: 10 per 1000 | | | | | | | | | | | | |
| 1 | RCT   (PRoFESS) | Not serious | Not serious | Not serious | Serious  Downgraded with 1 level due to confidence interval including both trivial effect and important harm | - | 426/8,607 (4.9%) | 391/8,663 (4.5%) | *Odds ratio: 1.10 (CI 95% 0.96 – 1.27)  Based on data from 17,270 participants from 1 RCT | **4 more** per 1,000  (from 2 fewer to 11 more) | ⨁⨁⨁◯ (Moderate) | Dual antithrombotic treatments in adults that experienced a stroke likely makes little to no difference on dementia incidence compared to monotreatment |
| Outcome 2 (binary): Mild cognitive impairment (≥ 1.5 SD below mean on ≥ 2 memory score) - Minimally important difference: 10 per 1000 | | | | | | | | | | | | |
| 1 | RCT   (SPS3) | Not serious | Not serious | Not serious | Very serious  Downgraded with 2 levels due to confidence interval including both important benefit and important harm | - | 189/721 (26.2%) | 187/692 (27.0%) | *Odds ratio: 0.96 (CI 95% 0.76 – 1.21)  Based on data from 1,413 participants from 1 RCT | **8 fewer** per 1,000  (from 51 fewer to 39 more) | ⨁⨁◯◯ (Low) | Dual antithrombotic treatments in adults that experienced a stroke may make little to no difference on mild cognitive impairment incidence compared to monotreatment |
| Outcome 3 (binary): Cognitive decline (based on Δ MMSE ≥ 3) - Minimally important difference: 10 per 1000 | | | | | | | | | | | | |
| 1 | RCT   (PRoFESS) | Not serious | Not serious | Not serious | Serious  Downgraded with 1 level due to confidence interval including both trivial effect and important harm | - | 764/7427 (10.3%) | 863/ 7622 (11.3%) | *Odds ratio: 0.90 (CI 95% 0.81 – 1.00)  Based on data from 15,049 participants from 1 RCT | **10 fewer** per 1,000  (from 19 fewer to 0 fewer) | ⨁⨁⨁◯ (Moderate) | Dual antithrombotic treatments in adults that experienced a stroke likely makes little to no difference on cognitive decline compared to monotreatment |
| Outcome 4 (binary): Fatal events** - Minimally important difference: 10 per 1000 | | | | | | | | | | | | |
| 1 | RCT (PRoFESS) | Not serious | Not serious | Not serious | Not serious | - | 739/10,181 (7.3%) | 756/10,151 (7.4%) | *Odds ratio: 0.97 (CI 95% 0.88 – 1.08)  Based on data from 20,332 participants from 1 RCT | **2 fewer** per 1,000    (from 8 fewer to 5 more) | ⨁⨁⨁⨁ (High) | Dual antithrombotic treatments in adults that experienced a stroke makes little to no difference on fatal events compared to monotreatment |
| Outcome 5 (binary): All SAEs*** | | | | | | | | | | | | |
| 1 | RCT  (PRoFESS) | Not serious | Not serious | Not serious | Serious  Downgraded with 1 level due to confidence interval including both trivial effect and important harm | - | 2,749/10,181 (27.0%) | 2,724/10,151 (26.8%) | *Odds ratio: 1.00 (CI 95% 0.95 – 1.07)  Based on data from 20,332 participants from 1 RCT | **0 fewer** per 1,000  (from 10 fewer to 13 more) | ⨁⨁⨁◯ (Moderate) | Dual antithrombotic treatments in adults that experienced a stroke likely makes little to no difference in SAEs compared to monotreatment |

*Odds ratio determined by self-conducted random-effects Mantel-Haenszel meta-analysis.

**Cause of death: cardiovascular causes, recurrent stroke, myocardial infarction, new or worsening heart failure.

*** SAEs: cardiac disorders, gastrointestinal disorders, general disorders, hepatobiliary disorders, infections, injury/poisoning, metabolism/nutrition disorders, musculoskeletal disorders, neoplasms, nervous system disorders, psychiatric disorders, renal and urinary disorders, respiratory and thoracic disorders, vascular disorders.

^1^4 categories of certainty of evidence: ⨁⨁⨁⨁ (High), ⨁⨁⨁◯ (Moderate), ⨁⨁◯◯ (Low), ⨁◯◯◯ (Very low). Examples are provided in the table.

^2^ For references regarding plan language summaries, please see: <https://training.cochrane.org/sites/training.cochrane.org/files/public/uploads/Nancy%20Santesso%20slides.pdf>; <https://www.sciencedirect.com/science/article/pii/S0895435619304160>

Abbreviations: CI, confidence interval; CASI, The Cognitive Abilities Screening Instrument; DSM IV, Diagnostic and Statistical Manual of Mental Disorders Revision IV; MMSE, Mini-Mental State Examination; RCT, randomized controlled trial; SAE, serious adverse event  event; SD, standard deviation

**GRADE table 4: Statins treatment vs no statins treatments in adults that experienced a non-cardioembolic ischemic stroke**.

Population: Adults that experienced a non-cardioembolic ischemic stroke

Intervention: Statins treatment

Comparator: No statins treatment

References:

Hosomi N, Nagai Y, Kohriyama T, Ohtsuki T, Aoki S, Nezu T, Maruyama H, Sunami N, Yokota C, Kitagawa K, Terayama Y, Takagi M, Ibayashi S, Nakamura M, Origasa H, Fukushima M, Mori E, Minematsu K, Uchiyama S, Shinohara Y, Yamaguchi T, Matsumoto M; J-STARS Collaborators. The Japan Statin Treatment Against Recurrent Stroke (J-STARS): A Multicenter, Randomized, Open-label, Parallel-group Study. EBioMedicine. 2015 Aug 6;2(9):1071-8. doi: 10.1016/j.ebiom.2015.08.006. PMID: 26501105; PMCID: PMC4588424.

| **Certainty assessment** | | | | | | | **№ of patients** | | **Effect** | | **Certainty^1^** | **Plain language summary^2^** |
| --- | --- | --- | --- | --- | --- | --- | --- | --- | --- | --- | --- | --- |
| **№ of studies** | **Study design** | **Risk of bias** | **Inconsistency** | **Indirectness** | **Imprecision** | **Other considerations** | **Interventions** | **Control** | **Relative  (95% CI)** | **Absolute  (95% CI)** |  |  |
| Outcomes 1 (binary): Incident dementia (based on DSM-IIIR) - Minimally important difference: 10 per 1000 | | | | | | | | | | | | |
| 1 | RCT (J-STARS) | Not serious | Not serious | Not serious | Very serious  Downgraded with 2 levels due to confidence interval including both important harm and important benefit | - | 32/780   (4.1%) | 33/785 (4.2%) | *Odds ratio: 0.97 (CI 95% 0.59 – 1.60)  Based on data from 1,565 participants from 1 RCT | **1 fewer** per 1,000  From 17 fewer to 24 more | ⨁⨁◯◯ (Low) | Statins treatment in adults that experienced a non-cardioembolic ischemic stroke may make little to no difference on dementia incidence compared to no statins treatment |
| Outcome 2 (binary): All SAEs** – Minimally important difference: 10 per 1000 | | | | | | | | | | | | |
| 1 | RCT  (J-STARS) | Not serious | Not serious | Not serious | Serious  Downgraded with 1 level due to confidence interval including both trivial effect and important harm | - | 188/780 (24.1%) | 164/785 (20.9%) | *Odds ratio: 1.20 (CI 95% 0.95 – 1.53)  Based on data from 1,565 participants from 1 RCT | **32 more** per 1,000  (from 8 fewer to 79 more) | ⨁⨁⨁◯ (Moderate) | Statins treatment in adults that experienced a non-cardioembolic ischemic stroke likely increases SAEs compared to no statins treatment |

*Odds ratio determined by self-conducted random-effects Mantel-Haenszel meta-analysis.

**SAEs: cancer, pneumonia, fracture, cataract, colon polyp, rhabdomyolysis, deviating laboratory results

^1^4 categories of certainty of evidence: ⨁⨁⨁⨁ (High), ⨁⨁⨁◯ (Moderate), ⨁⨁◯◯ (Low), ⨁◯◯◯ (Very low). Examples are provided in the table.

^2^ For references regarding plan language summaries, please see: <https://training.cochrane.org/sites/training.cochrane.org/files/public/uploads/Nancy%20Santesso%20slides.pdf>; <https://www.sciencedirect.com/science/article/pii/S0895435619304160>

Abbreviations: DSM IIIR, Diagnostic and Statistical Manual of Mental Disorders Revision III; MMSE, Mini-Mental State Examination; RCT, randomized controlled trial; SAE, serious adverse event

**GRADE table 5: Stricter lipid-lowering guidelines vs regular lipid-lowering guidelines in adults that experienced an ischemic or haemorrhagic stroke**.

Population: Adults that experienced an ischemic or haemorrhagic stroke

Intervention: Strict lipid-lowering guidelines

Comparator: Regular lipid-lowering guidelines

References:

Bath PM, Scutt P, Blackburn DJ, Ankolekar S, Krishnan K, Ballard C, Burns A, Mant J, Passmore P, Pocock S, Reckless J, Sprigg N, Stewart R, Wardlaw JM, Ford GA; PODCAST Trial Investigators. Intensive versus Guideline Blood Pressure and Lipid Lowering in Patients with Previous Stroke: Main Results from the Pilot 'Prevention of Decline in Cognition after Stroke Trial' (PODCAST) Randomised Controlled Trial. PLoS One. 2017 Jan 17;12(1):e0164608. doi: 10.1371/journal.pone.0164608. PMID: 28095412; PMCID: PMC5240987.

| **Certainty assessment** | | | | | | | **№ of patients** | | **Effect** | | **Certainty^1^** | **Plain language summary^2^** |
| --- | --- | --- | --- | --- | --- | --- | --- | --- | --- | --- | --- | --- |
| **№ of studies** | **Study design** | **Risk of bias** | **Inconsistency** | **Indirectness** | **Imprecision** | **Other considerations** | **Interventions** | **Control** | **Relative  (95% CI)** | **Absolute  (95% CI)** |  |  |
| Outcome 1 (continuous): Cognitive decline (based on Mean Δ MMSE) - Minimally important difference: 3 points MD | | | | | | | | | | | | |
| 1 | RCT (PODCAST) | Not serious | Not serious | Not serious | Very serious  Downgraded with 2 levels due to very small sample size and due to confidence interval including both trivial effect and important benefit | - | Group size = 39 | Group size = 38 | Mean difference: **1.9 points higher** (higher means better; CI 95% 0.3 points lower to 4.1 points higher)  Based on data from 77 participants in 1 RCT | | ⨁⨁◯◯ (Low) | Stricter lipid-lowering guidelines in adults that experienced an ischemic or haemorrhagic stroke may make little to no difference on cognitive decline compared to regular lipid-lowering guidelines. |
| Outcome 2 (continuous): Quality of life (based on Δ EQ-VAS) - Minimally important difference: 10 points MD | | | | | | | | | | | | |
| 1 | RCT (PODCAST) | Not serious | Not serious | Not serious | Very serious  Downgraded with 2 levels due to very small sample size and due to confidence interval including both trivial effect and important benefit | - | Group size = 39 | Group size = 38 | Mean difference: **8.6 points higher** (higher means better; CI 95% 1.7 points higher to 15.5 points higher)  Based on data from 77 participants in 1 RCT | | ⨁⨁◯◯ (Low) | Stricter lipid-lowering guidelines in adults that experienced an ischemic or haemorrhagic stroke may make little to no difference on quality of life compared to regular lipid-lowering guidelines. |
| Outcome 3 (continuous): ADL (based on Δ Barthel Index) - Minimally important difference: 10 points MD | | | | | | | | | | | | |
| 1 | RCT (PODCAST) | Not serious | Not serious | Not serious | Very serious  Downgraded with 2 levels due to very small sample size and due to confidence interval including both trivial effect and important benefit |  | Group size = 39 | Group size = 38 | Mean difference: **7.5 points higher** (higher means better; CI 95% 0.5 points lower to 15.4 points higher)  Based on data from 77 participants in 1 RCT | | ⨁⨁◯◯ (Low | Stricter lipid-lowering guidelines in adults that experienced an ischemic or haemorrhagic stroke may make little to no difference on ADL compared to regular lipid-lowering guidelines. |
| Outcome 4 (binary): Fatal events** - Minimally important difference: 10 per 1000 | | | | | | | | | | | | |
| 1 | RCT (PODCAST) | Not serious | Not serious | Not serious | Extremely serious  Downgraded with 3 levels due to very small sample size and due to confidence interval including both important benefit and important harm | - | 3/39 (7.7%) | 4/38 (10.5%) | *Odds ratio: 0.71 (CI 95% 0.15 – 3.40)  Based on data from 77 participants in 1 RCT | **28 fewer** per 1,000  (from 88 fewer to 180 more) | ⨁◯◯◯ (Very low) | The evidence is very uncertain about the effect of stricter lipid-lowering guidelines in adults that experienced an ischemic or haemorrhagic stroke on fatal events compared to regular lipid-lowering guidelines. |
| Outcome 5 (binary): All SAEs*** – Minimally important difference: 10 per 1000 | | | | | | | | | | | | |
| 1 | RCT (PODCAST) | Not serious | Not serious | Not serious | Extremely serious  Downgraded with 3 levels due to very small sample size and due to confidence interval including both important benefit and important harm | - | 17/ 39 (43.6%) | 21/38 (55.3%) | *Odds ratio: 0.63 (CI 95% 0.25 – 1.54)  Based on data from 77 participants in 1 RCT | **115 fewer** per 1,000  (from 717 fewer to 103 more) | ⨁◯◯◯ (Very low) | The evidence is very uncertain about the effect of stricter lipid-lowering guidelines in adults that experienced an ischemic or haemorrhagic stroke on SAEs compared to regular lipid-lowering guidelines. |

*Odds ratio determined by self-conducted random-effects Mantel-Haenszel meta-analysis.

**Cause of death: dementia, stroke, sudden cardiac death, death unattended, tumour – malignant, pneumonia.

*** SAEs: cardiovascular, genotourinary, gastrointestinal, haematological, metabolic, musculoskeletal, nervous system, respiratory, other

^1^4 categories of certainty of evidence: ⨁⨁⨁⨁ (High), ⨁⨁⨁◯ (Moderate), ⨁⨁◯◯ (Low), ⨁◯◯◯ (Very low). Examples are provided in the table.

^2^ For references regarding plan language summaries, please see: <https://training.cochrane.org/sites/training.cochrane.org/files/public/uploads/Nancy%20Santesso%20slides.pdf>; <https://www.sciencedirect.com/science/article/pii/S0895435619304160>

Abbreviations: CI, confidence interval; DSM IV, Diagnostic and Statistical Manual of Mental Disorders Revision IV; EQ-VAS, EuroQol visual analogue scale MMSE, Mini-Mental State Examination; RCT, randomized controlled trial; SAE, serious adverse even
